# Selection Bias When Estimating Average Treatment Effects Using One-sample Instrumental Variable Analysis

**DOI:** 10.1097/EDE.0000000000000972

**Published:** 2019-04-08

**Authors:** Rachael A. Hughes, Neil M. Davies, George Davey Smith, Kate Tilling

**Affiliations:** From the a Population Health Sciences, Bristol Medical School, University of Bristol, Bristol, United Kingdom; b MRC Integrative Epidemiology Unit, University of Bristol, Bristol, United Kingdom.

**Keywords:** Causal exposure effect, Collider stratification bias, Instrumental variable, Selection bias, Two-stage least squares

## Abstract

Participants in epidemiologic and genetic studies are rarely true random samples of the populations they are intended to represent, and both known and unknown factors can influence participation in a study (known as selection into a study). The circumstances in which selection causes bias in an instrumental variable (IV) analysis are not widely understood by practitioners of IV analyses. We use directed acyclic graphs (DAGs) to depict assumptions about the selection mechanism (factors affecting selection) and show how DAGs can be used to determine when a two-stage least squares IV analysis is biased by different selection mechanisms. Through simulations, we show that selection can result in a biased IV estimate with substantial confidence interval (CI) undercoverage, and the level of bias can differ between instrument strengths, a linear and nonlinear exposure–instrument association, and a causal and noncausal exposure effect. We present an application from the UK Biobank study, which is known to be a selected sample of the general population. Of interest was the causal effect of staying in school at least 1 extra year on the decision to smoke. Based on 22,138 participants, the two-stage least squares exposure estimates were very different between the IV analysis ignoring selection and the IV analysis which adjusted for selection (e.g., risk differences, 1.8% [95% CI, −1.5%, 5.0%] and −4.5% [95% CI, −6.6%, −2.4%], respectively). We conclude that selection bias can have a major effect on an IV analysis, and further research is needed on how to conduct sensitivity analyses when selection depends on unmeasured data.

## Abstract 2

Supplemental Digital Content is available in the text.

The main aim of many epidemiologic studies is to estimate the causal effect of an exposure on an outcome. Instrumental variable (IV) analyses are increasingly used to overcome bias owing to unmeasured confounding. An IV analysis requires a variable, known as the instrument, to satisfy three assumptions: the instrument is associated with the exposure, the instrument only causes the outcome to change via its impact on the exposure, and there is no confounding between the instrument and the outcome.^1–3^ Based on the observed data, the first IV assumption can be tested, but the latter two are unverifiable.^4^

As with any statistical analysis, inference about the causal exposure effect (here onwards, shortened to exposure effect) may be invalid when the sample included in the analysis is not a representative (random) sample of the target population. This could be due to selection into the study, participant dropout, loss to follow-up, subgroup analysis, or missing data. There may be both known and unknown factors that influence the “selection” of participants for analysis.

Following Hernán and Robins,^5^ we consider selection bias to be distinct from confounding. Confounding is attributable to the presence of common causes of the outcome and exposure. In contrast, selection bias is attributable to conditioning on common effects (e.g., of the outcome and exposure) and is a type of collider-stratification bias.^6,7^ The IV estimate of the exposure effect in the study sample is biased by selection when it systematically differs to the value of the exposure effect in the target population.^8^ Selection bias is concerned with the internal validity of a study, as opposed to external validity (using a study’s results to make inferences about populations that differ from the target population).^9–11^ Internal validity is essential before external validity can be considered.

Although the methodologic literature recognizes that IV analyses do not protect against selection bias,^5,6,12–21^ it is seldom acknowledged in IV analyses^22^ or discussed in guidelines for IV analysis.^23–28^

In the IV literature, a small number of studies have used directed acyclic graphs (DAGs)^29–32^ to illustrate when selection violates the assumptions of an IV analysis.^15–18^ However, these studies cover a limited range of selection scenarios, with Gkatzionis and Burgess^15^ confining their discussion to Mendelian randomization, and Ertefaie et al.^17^ and Canan et al.^18^ provide an incomplete explanation of the consequence of selection. Only one study^15^ considered if the effects of selection differed according to a null and non-null exposure effect, and none of these papers investigated whether the consequences of selection differed according to a linear and nonlinear exposure–instrument association.

We use DAGS to illustrate the circumstances in which an IV analysis is biased by selection for a wide range of selection scenarios. Through simulations, we show how the consequences of selection can depend on the factors determining selection, strength of the instrument, whether the causal effect is null or not null, and linearity of the exposure–instrument association. Using a real application, we show how an IV analysis ignoring nonrandom selection can reach different conclusions to an IV analysis which adjusts for nonrandom selection.

## WHEN DOES SELECTION LEAD TO BIAS?

### Description of Our IV Analysis

We want to estimate the effect of a continuous exposure 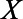 on a continuous outcome 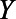, and we denote this exposure effect by 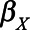. The 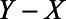 association is confounded by unmeasured variables 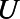 and measured variables 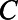. In the full sample (selected and unselected participants), the instrument 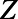 satisfies the three IV assumptions (without conditioning on 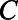).

To identify 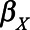, we assume homogeneous exposure effects (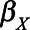 is the same for all individuals^26^). We estimate 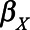 using the two-stage least squares method^33^ and denote its two-stage least squares estimate by 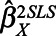. In the first stage of two-stage least squares, 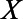 is regressed on 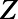 to give fitted values 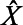. In the second stage, the regression coefficient of 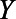 on fitted values 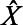 is the two-stage least squares estimate, 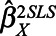. When 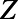 is a single instrument, 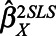 is equivalently estimated using the ratio of coefficients method.^34,35^


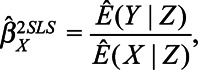
(1)

where the numerator 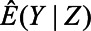 is the estimated coefficient from the regression of 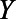 on 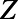, and the denominator 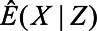 is the estimated coefficient from the regression of 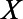 on 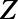. We also estimate the exposure effect conditional on measured confounders 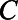 and denote this conditional two-stage least squares estimate by 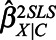.

### Selection Mechanisms

Whether 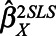 is biased by selection depends on the reasons for selection (the “selection mechanism”). We discuss 10 out of the 32 possible selection mechanisms for our IV example. The remaining selection mechanisms can be explained using one or a combination of the 10 described below. Also, we chose selection mechanisms partially dependent on 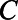 and not 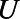 because we wanted to illustrate when a conditional IV analysis does and does not remove bias because of measured confounders influencing selection.

**FIGURE 1.**
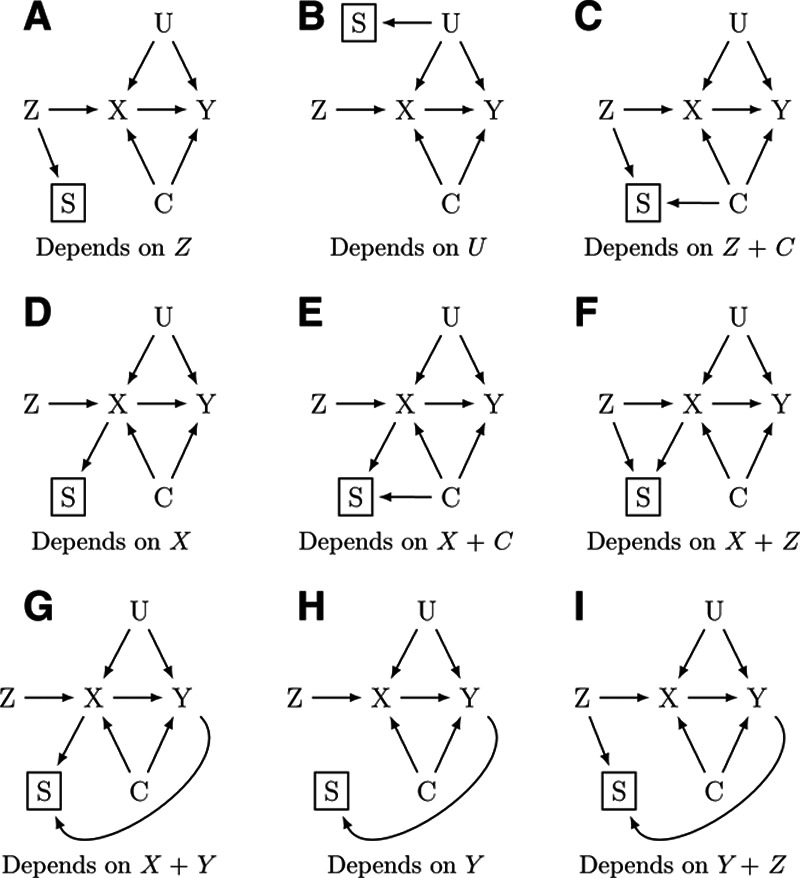
Directed acyclic graphs of an instrumental variable analysis under nine different selection mechanisms. Panels A to I correspond to selection depending on Z, U, Z + C, X, X + C, X + Z, X + Y, Y, and Y + Z, respectively.

Figure 1A-I depict DAGs showing the causal relationships among the variables of our IV analysis under different selection mechanisms, where 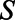 is a binary variable indicating whether a participant is selected or unselected. Restricting the analysis to the selected sample implies conditioning on 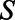, which is represented by a box around 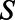. Because a DAG is nonparametric, the discussion below is not specific to continuous variables only. Unless otherwise stated, whether 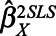 is biased by selection equally applies when the true causal effect is null and not null. Also, in our example all variables are measured without error; however, whether 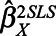 is biased by selection equally applies when selection depends on variables measured with error.^5^

Table 1 summarizes when 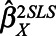 and 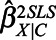 are biased by selection for these 10 selection mechanisms. When selection is completely at random, or depends on 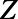 (Figure 1A) or 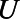 (Figure 1B), 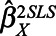 and 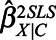 are not biased by selection. Here, the IV assumptions remain true in the selected sample (e.g., all pathways between 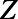 and 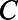 or 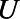 remain blocked by a collider and so the 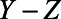 association is unconfounded in the selected sample).

**TABLE 1.**
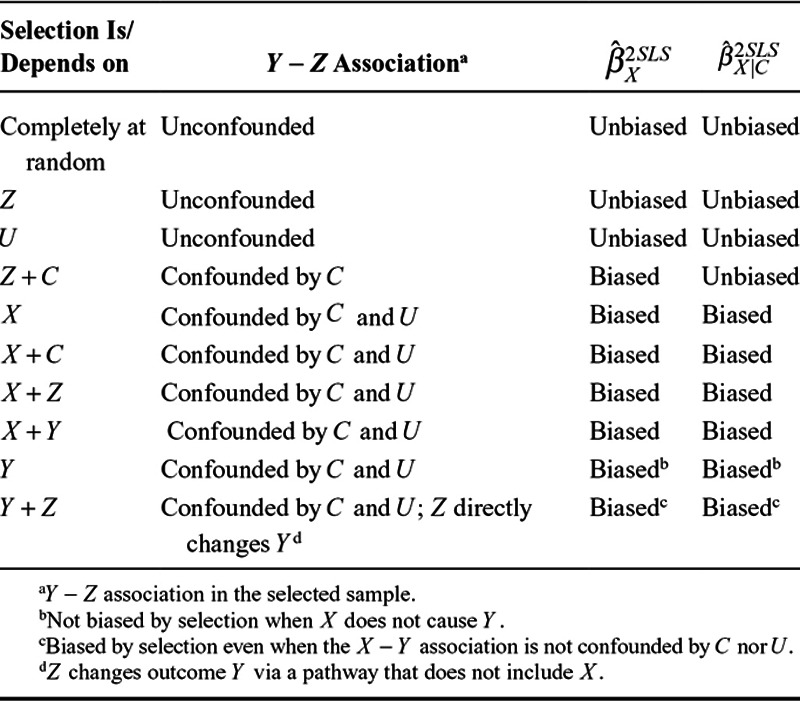
Potential Bias of the Two-stage Least Squares Estimate of the Causal Exposure Effect, 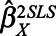, and the Corresponding Two-stage Least Squares Estimate Conditional on C, 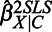, According to Different Selection Mechanisms

When selection depends on 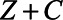, 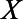, 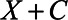, 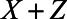, 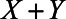, or 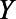 (Figure 1C–H, respectively), 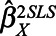 is biased by selection because the 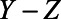 association becomes confounded in the selected sample. Selection implies conditioning on a collider (or a descendant of a collider as per selection on 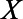 or 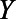), which opens a noncausal pathway between 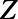 and 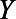 via a confounder (e.g., selection on 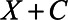 opens pathway 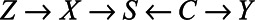). For selection mechanism 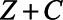, the 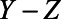 association is confounded by 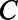 only. Therefore, while 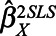 is biased by selection on 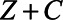, 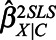 is not biased because the only noncausal pathway is via 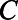, which is reblocked by conditioning on 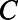. For the other selection mechanisms, the 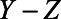 association is confounded by 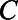 and 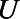. Therefore, while estimating 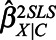 reduces the level of selection bias (by eliminating confounding by 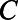), 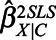 remains biased because the 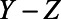 association is still confounded by 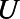 in the selected sample.

Selection depending on 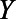 has the special property that 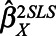 and 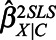 are only biased by selection when 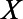 causes 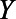 (the true exposure effect is not null). When 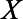 does not cause 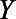, the pathways between 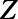 and 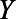 via 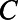 and 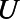 are blocked by the absence of an edge between 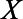 and 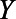 (e.g., 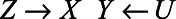).

When selection depends on 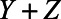 (Figure 1I), 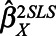 and 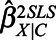 are biased by selection because the instrument is directly associated with the outcome in the selected sample. Selection implies conditioning on collider 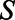, which unblocks pathway 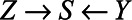. When 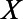 causes 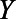, selection depending on 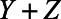 also results in violating a second IV assumption because the 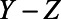 association is confounded by 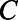 and 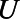 in the selected sample (as discussed for selection on 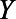 only).

Further information is given in section “Detailed discussion on selection mechanisms” in the eAppendix; http://links.lww.com/EDE/B499.

## SIMULATION STUDY

We investigated the effects of different selection mechanisms on 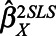 for the above IV analysis. For the sake of brevity, we only included two of the three selection mechanisms that do not bias 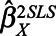 and 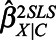, thereby excluding selection on 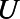.

### Methods

We simulated data on 
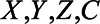
, and 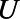 under a multivariate normal distribution, with the relationships among these variables as depicted in Figure 1 (e.g., assuming independence between 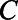 and 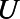). We ensured the three IV assumptions held true in the full sample.

Selection was imposed using a logistic regression model, where the covariates of the model included one or more of 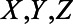, and 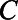 (depending on the selection mechanism). For all selection mechanisms, close to 60% of the participants were selected. We used Stata (Stata Corp, Texas) command *ivregress* to perform two-stage least squares estimation. We also conducted a weighted two-stage least squares analysis, using inverse probability weighting (IPW),^36^ in which the weights try to make the selected participants a representative sample of the study population.^17^

We repeated the simulation study for: (1) a causal exposure effect of 1 and a noncausal exposure effect (mean difference of 1 and 0, respectively), (2) a strong instrument (partial 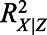 close to 0.39 in the full sample) and a moderate instrument (partial 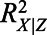 close to 0.045 in the full sample, and (3) a linear 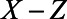 association (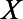 as a function of 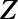) and a nonlinear 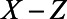 association (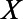 as a function of 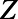 and 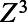). For all combinations of the simulation settings, we generated 3000 simulated data sets, each with 20,000 participants for the full sample.

Of interest was the bias of 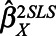, relative error of its standard error compared to the empirical SD of 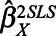, and coverage of the 95% confidence interval (CI) for 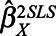. Similarly, for 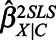. Evidence of systematic bias (estimates systematically differ from the true value) occurs when the Monte Carlo 95% CI for the bias (bias ± 1.96 × Monte Carlo standard error) excludes zero. Also, based on 3000 simulations, the Monte Carlo standard error for the true coverage percentage of 95 is 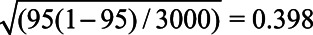,^37^ implying that the estimated coverage percentage should lie within the range of 94.2 and 95.8 (with 95% probability). We analyzed the simulation results using the *simsum* command.^38^

### Results

**FIGURE 2.**
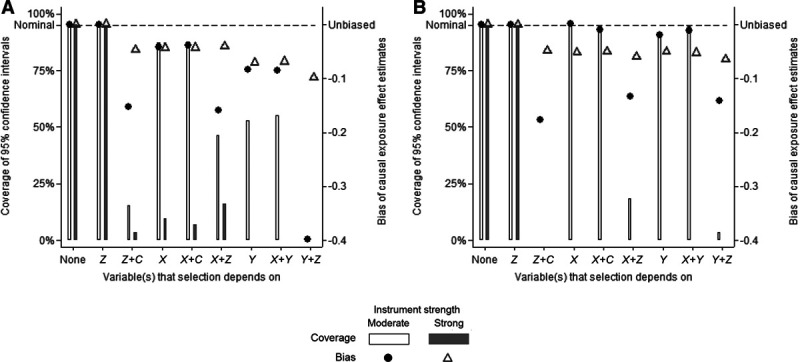
Bias of the two-stage least squares estimates (scatter points; right *y* axis), and coverage of their 95% CIs (bars; left *y* axis) according to different selection mechanisms and instrument strengths: moderate and strong. A and B correspond to linear and nonlinear exposure–instrument association, respectively. The true value of the causal exposure effect was 1.

When there was no selection (the full sample), 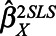 was unbiased and CI coverage was nominal (close to 95%) in all cases (eTables 3–6; http://links.lww.com/EDE/B499). Figure 2 shows the bias of the two-stage least squares estimates (scatter points; right *y* axis) and CI coverage (bars; left *y* axis) according to the nine selection mechanisms and instrument strengths moderate and strong, when the true exposure effect was 1: Figure 2A, B correspond to linear and nonlinear 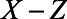, respectively. Full results are reported in eTables 3 and 4; http://links.lww.com/EDE/B499.

When selection was completely at random (represented as “none”) or depended on 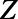 only, 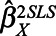 was unbiased and CI coverage was nominal. Because this finding applied to all simulation settings, we shall not discuss these two selection mechanisms further. For the remaining selection mechanisms, 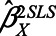 was negatively biased with moderate (88%) to severe (0%) CI undercoverage (shown by the absence of a bar) for linear 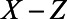 (Figure 2A).

For linear and nonlinear 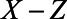, selection depending on 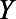 did not bias 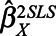 when the exposure effect was null (eTables 5 and 6; http://links.lww.com/EDE/B499). For the remaining selection mechanisms, the results were very similar for a causal and noncausal exposure effect.

#### Impact of Instrument Strength

When selection partly depended on 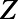 (selection mechanisms 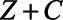, 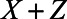, and 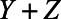), the level of bias increased with decreasing instrument strength. Otherwise, there were only small differences in the level of bias between the instrument strengths. For all selection mechanisms, standard errors were larger for the weaker instrument, which mostly resulted in higher CI coverage.

#### Nonlinear Versus Linear 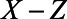 Association

Generally, the nonlinear 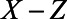 results (Figure 2B) follow the same patterns noted for linear 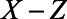. Differences in the level of bias between linear and nonlinear 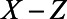 were far larger for the moderate instrument than the strong instrument because (owing to the simulation study design) the strength of the nonlinearity was the same for the moderate and strong instruments.

For selection mechanism 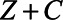, the effect of the nonlinearity was to decrease the instrument strength, thus increasing the level of bias: when the instrument was moderate, the level of bias was 15% higher and the instrument strength (partial 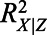) was 17% lower for nonlinear 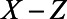 compared with linear 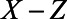 (eTable 4; http://links.lww.com/EDE/B499). Conversely, for selection mechanism 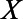, when the instrument was moderate, the level of bias was 36 times smaller for nonlinear 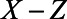 compared with linear 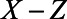. Nonlinearity caused a large change in the distribution of 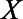 among the selected participants, and this change in the distribution weakened the induced 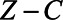 and 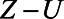 associations, and hence the large reduction in bias. A similar pattern was noted for the other selection mechanisms depending on 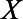 or a descendant of 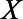.

For the moderate and strong instruments, the standard errors of 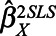 were smaller for nonlinear 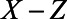 than linear 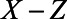. Consequently, when the level of bias was comparable between linear and nonlinear 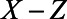, CI coverages were poorer for nonlinear 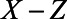 owing to the smaller standard errors. However, where nonlinearity lowered the level of bias (e.g., selection on 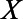), then CI coverages were higher for nonlinear 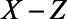 despite smaller standard errors.

#### Exposure Effect Conditional on 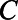


For selection mechanism 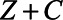, 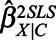 was unbiased and CI coverage was nominal for all simulation settings (eTables 8–11; http://links.lww.com/EDE/B499). For the remaining mechanisms, the level of bias for 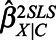 was between 18% and 73% lower than that of 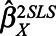, and CI coverages for 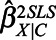 were up to 3.8 times higher. Otherwise, the results for 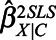 follow the same patterns noted for 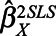.

#### Weighted Two-stage Least Squares Analysis

For linear 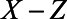, the weighted analyses gave unbiased estimates of 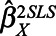 with nominal CI coverages for all selection mechanisms (eTable 12; http://links.lww.com/EDE/B499). However, for nonlinear 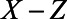, there was a small amount of systematic bias and CI undercoverage around 90% for all selection mechanisms, except 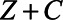, which was attributable to inflated weights (eTable 13; http://links.lww.com/EDE/B499).

## APPLIED EXAMPLE

We conducted an IV analysis to ascertain whether leaving school before the age of 16 years had a causal effect on the decision to smoke^23^ using data from the UK Biobank study,^39^ where there is evidence of nonrandom selection.^40^ See section “Detailed description of the applied example” in the eAppendix; http://links.lww.com/EDE/B499 for further information.

The binary outcome 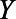 was equal to one for ever smokers (included ex-smokers and current smokers) and equal to zero for never smokers. We also considered a second binary outcome, equal to one for current smokers and equal to zero for ex-smokers and never smokers. We performed separate analyses on each outcome using the same exposure and instrument. The binary exposure 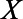 was equal to one if the participant had left school aged 16 years or older, and equal to zero otherwise. We used a policy reform (called ROSLA, Raising of School Leaving Age) as an instrument for time spent in education. The binary instrument 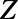 was equal to one if the participant turned 15 years old after the policy reform was introduced, and equal to zero otherwise. Also, there were some measured confounders, 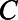, of the exposure–outcome association (e.g., sex, month of birth) but we suspected many unmeasured confounders, 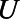.

The UK Biobank study is a sample of 502,644 UK residents enrolled between 2006 and 2010.^39^ The study response rate was 5.5%, and higher levels of educational achievement predicted participation.^23^ This suggests that the study participants were selected depending on 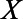, educational attainment, which can bias an IV analysis.

To maximize the plausibility of the IV assumptions, we restricted our analysis to participants who turned 15 years old within the period of 1 year before to 1 year after the introduction of the ROSLA policy.

We performed two-stage least squares estimation using the linear probability model, where the exposure effect is on the risk difference scale.^41^ We calculated robust standard errors to account for assumptions about homogeneous exposure effects and the outcome distributions. We also considered the equivalent standard analysis: the linear regression of 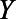 on 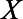 along with the same measured confounders as the IV analysis (with robust standard errors). This example is for illustrative purposes only; in practice, one would include all available measured confounders in the linear regression analysis. Although a linear regression may be biased by unmeasured confounding, its exposure effect estimate is not biased by selection on 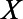.^42^

We used IPW to account for selection on educational achievement; thus, the weighted IV analysis accounts for unmeasured confounding and nonrandom selection. We generated the weights under the assumption that selection only depended on 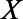 (see section “Calculation of the weights” in the eAppendix; http://links.lww.com/EDE/B499 for further information). Those participants suspected to be underrepresented in the selected sample (left school aged 15 years) had larger weights, and hence contributed more to the weighted analysis, than those suspected to be overrepresented in the selected sample (left school aged 16 years or older). For completeness, we carried out a weighted linear regression analysis using the same weights, even though weighting should have no effect because selection on 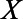 would not cause bias.

Table 2 presents the results, based on 22,138 participants, for the exposure effect estimated using unweighted and weighted versions of linear regression and IV analysis. For the IV analysis, there were noticeable differences between the unweighted and weighted analyses. For outcome “ever smoker,” the weighted IV estimate was more than double than that of the unweighted IV estimate, although there was some overlap between the corresponding 95% CIs. Both analyses suggested staying in school at least 1 extra year decreased the likelihood of being an ever smoker compared with those who left school at the age of 15 years, although the CI for the unweighted analysis was inconclusive because it included all three possible conclusions: risk decrease, no effect, and risk increase. For outcome “current smoker,” the results of the unweighted IV analysis, risk difference 1.8% (95% CI, −1.5%, 5.0%), suggested staying in school at least 1 extra year increased the likelihood of being a current smoker compared with those who left school aged 15 years, while the results of the weighted IV analysis, −4.5% (95% CI, −6.6%, −2.4%), suggested the opposite effect. The CI for the unweighted IV analysis was inconclusive. As expected, the unweighted and weighted linear regression results were identical.

**TABLE 2.**
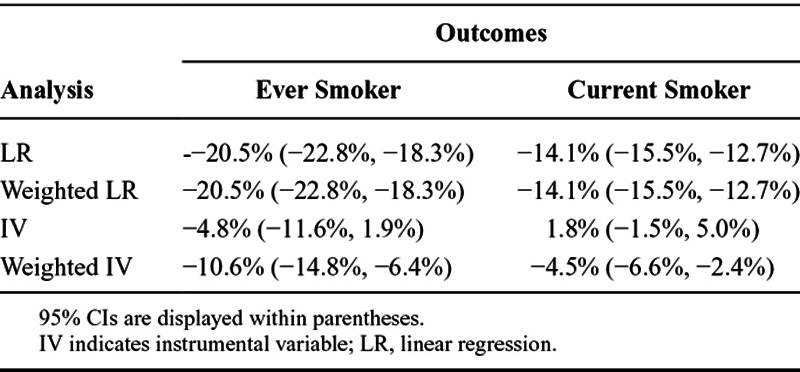
Risk Difference %, of Ever Smoker or Current Smoker, for Leaving School at the Age of 16 Years or Older Compared with Leaving School at the Age of 15 Years Using Unweighted and Weighted Versions of LR and IV Analysis

Comparing the analyses which should not be biased by selection on 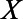, the linear regression exposure effect estimates were about two to three times larger than those of the weighted IV, and there was no overlap in the 95% CIs. These differences may be due to the presence of unmeasured confounding, which would only bias the linear regression analyses. However, other possible causes of the differences include an instrument that does not satisfy the IV analysis assumptions or heterogeneous treatment effects.

We also conducted a simulation study based on this example where the instrument, exposure, and outcome were binary, and we investigated the effects of different selection mechanisms on 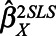. The results followed the same patterns noted for our multivariate normal simulation study. See section “Simulation study based on the applied example” in the eAppendix; http://links.lww.com/EDE/B499 for further information.

## DISCUSSION

For 10 selection mechanisms, we have explained the structure of the selection bias and showed how DAGs can be used to determine whether selection violates any of the IV assumptions. The IV estimate of the exposure effect is not biased by selection when the selection is completely at random, depends only on the instrument, or depends only on confounders. For the remaining selection mechanisms, we have illustrated, using simulations, that nonrandom selection can result in a biased IV estimate and CI undercoverage. For a causal and null exposure effect, the IV estimate was biased, with often poor to severe CI undercoverage, when selection depended on the instrument plus measured confounder, or depended (in part or entirely) on the exposure, or the outcome plus exposure, or the outcome plus instrument. A special case was selection depending on the outcome only, where the IV estimate was only biased when 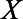 truly caused 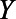. Decreasing the instrument strength resulted in an increase in the level of bias for selection mechanisms partly depending on the instrument, but had little effect on the other selection mechanisms. For all selection mechanisms, CI coverages were noticeably higher for the moderate instrument compared with the strong instrument because standard errors increased with decreasing instrument strength. Although the larger standard errors improved CI coverage, there was still substantial CI undercoverage. Estimating the conditional IV estimate eliminated selection bias when caused by measured confounding, but only reduced the level of bias when selection resulted in measured and unmeasured confounding. Changing the exposure–instrument association from linear to nonlinear reduced the size of the standard errors, but its effect on bias depended on the structure of the selection bias.

In keeping with the results of our simulation study, nontrivial levels of selection bias were demonstrated via simulations.^15,16,18–21^ Gkatzionis and Burgess^15^ investigated two selection mechanisms in the context of Mendelian randomization, and the remaining studies only considered a specific selection scenario.

Our study and others (e.g., Canan et al.^18^) assumed homogeneous exposure effects, but selection bias has also been described in an IV analysis that identifies the exposure effect in a subset of the population under the monotonicity assumption (e.g., Ertefaie et al.^17^).

Nonrandom selection can occur in practice, with large differences in the characteristics of the selected and unselected participants. For example, the percentage of subjects who owned their property outright was 56.7% in the UK Biobank study (the selected sample) and 40.6% in the 2001 UK census (the study population),^40^ so the odds of selection among outright property owners were almost double than that of those who were not outright property owners. Using similar calculations for the Avon Longitudinal Study of Parents and Children study,^43^ the odds of selection among households with a car was almost double the odds of selection among households without a car.

Our simulation study has several limitations. First, while we considered 10 plausible selection mechanisms, it was not possible to investigate all possible selection mechanisms even for a single IV analysis example. Second, in practice, an IV analysis may use weaker instruments than we considered. We chose a sample size that was typical of an IV analysis so that even for a partial 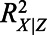 of 0.045, the instrument would not be considered weak. However, for the purposes of our study, we wanted to ensure that any bias was attributable only to selection and not to weak instrument bias.^35^ Third, our simulation study was designed to show the effects of different selection mechanisms on an IV analysis and not an exhaustive investigation of the levels of selection bias that could occur in practice. Fourth, our use of nonparametric DAGs, to determine if selection would violate one of the core IV assumptions, is not suitable for all types of selection mechanisms (e.g., when the occurrence of selection bias depends on the parameterization of the IV analysis^8^).

Some selection mechanisms bias the IV estimate but not the usual regression estimate, and in some situations, the selection bias of the IV estimate may exceed the confounding bias of the usual regression estimate. A larger bias for the IV estimate may also occur when both analyses are affected by nonrandom selection; e.g., in our main simulation study, for a moderate instrument with linear exposure–instrument association and causal exposure effect, selection on the outcome plus instrument resulted in a bias of −0.399 for the IV estimate but only 0.159 for the regression estimate.

IPW can account for nonrandom selection but may be unsuitable when individuals have large weights, and even IPW with weight trimming may be unsuitable.^15^ Although IPW usually requires selection to depend on measured data, other approaches have been proposed for selection depending on unmeasured or partially observed data.^17,19,20^

With individual-level data on the selected and unselected participants, an IV analyst can investigate possible factors that influence selection. However, this is impossible when the IV analyst only has summary-level data. Providers of summary-level data should discuss whether the study sample is a nonrandom sample of the target population and posit possible selection mechanisms. Where possible, these providers could generate summary-level data accounting for nonrandom selection (e.g., summary-level data from a weighted analysis or summary-level data adjusted for known factors associated with selection). Two-sample IV analyses tend to be conducted using summary-level data, and these analyses are further complicated because there are two opportunities for nonrandom selection to occur, and possibly two different selection mechanisms to take into account.

In summary, ignoring how participants are selected for analysis can result in a biased IV estimate and substantial CI undercoverage and can lead to an incorrect conclusion that an exposure is or is not causal. Although this limitation is beginning to be recognized in IV analysis guidelines for medical researchers,^22,44^ it needs to be more widely noted to ensure future published IV analyses routinely take into consideration possible bias owing to nonrandom selection. DAGs can be used to assess if the IV analysis may be biased by the assumed selection mechanism. Future work could provide researchers guidance on statistical methods, diagnostic tools, and sensitivity analyses for estimating causal effects from nonrandom samples.

## ACKNOWLEDGMENTS

We thank Frank Windmeijer, School of Economics, University of Bristol, for his helpful comments. This research has been conducted using the UK Biobank Resource under Application No. 8786.

## Supplementary Material
