# Supplementary material for: Selection Bias When Estimating Average Treatment Effects Using One-sample Instrumental Variable Analysis
Source: Epidemiology. 2019 Apr 8;30(3):350–7. doi: 10.1097/EDE.0000000000000972 (PMC6525095; doi:10.1097/EDE.0000000000000972)
Supplement: Supplementary file 1 [file ede-30-350-s001.pdf]

eAppendix: Selection bias when estimating average treatment effects using one-sample instrumental variable analysis

Detailed discussion on selection mechanisms

Selection completely at random, or depending on Z or U

When selection is completely at random, or selection depends on Z (Figure 1a), or selection depends on U (Figure 1b), $\hat{\beta}_X^{2SLS}$ is not biased by selection. **Explanation:** Selection does not imply conditioning on a collider (nor a descendant of a collider). The IV assumptions remain true in the selected sample; for example, within the selected sample, Z is not associated with C nor U because pathways $Z \rightarrow X \leftarrow U$ and $Z \rightarrow X \leftarrow C$ remain blocked by collider X, and pathways $Z \rightarrow X \rightarrow Y \leftarrow U$ and $Z \rightarrow X \rightarrow Y \leftarrow C$ remain blocked by collider Y. Therefore, the $Y - Z$ association is not confounded by C nor U in the selected sample.

Selection depending on Z and C

When selection depends on $Z + C$ (i.e., Z and C; Figure 1c), $\hat{\beta}_X^{2SLS}$ is biased because the $Y - Z$ association is confounded by C in the selected sample. **Explanation:** Selection implies conditioning on collider S which opens the noncausal pathway $Z \rightarrow \boxed{\text{S}} \leftarrow C \rightarrow Y$ [1, 2], and hence $Y - Z$ is confounded by C in the selected sample. Note, the $Y - Z$ association is not confounded by U in the selected sample (i.e., Z remains independent of U) because all pathways between Z and U remain blocked by a collider (e.g., $Z \rightarrow X \leftarrow U$). **Additional note:** Interestingly, conditioning on C re-blocks the aforementioned noncausal pathway via S, thus eliminating the selection bias. Therefore, whilst $\hat{\beta}_X^{2SLS}$ is biased by selection, the two-stage least squares estimate conditional on C, $\hat{\beta}_{X|C}^{2SLS}$, is not biased by selection. We note one exception. Suppose selection depends on $Z + C$ but we have measured confounder C

with error, denoted by C^* (DAG not shown). Conditioning on this mismeasured variable C^* would not block the noncausal pathway between Z and Y via S and C, and so selection bias would not be eliminated; that is, $\hat{\beta}^{2SLS}_{X|C}$ would remain biased by selection. Furthermore, conditioning on C^* would result in confounding the $Y - Z$ association by the measurement error of C, known as "residual confounding".

Selection depending on X

When selection depends on X, $X + C$, $X + Z$ or $X + Y$ (Figures 1d, 1e, 1f or 1g, respectively), $\hat{\beta}^{2SLS}_{X}$ is biased by selection due to confounding of the $Y - Z$ association by C and U.
Explanation: Because S is a descendant of X, conditioning on S unblocks pathways which include X as a collider (i.e., $Z \rightarrow X \leftarrow C$ and $Z \rightarrow X \leftarrow U$) [1, 2]; thus, Z is associated (i.e., conditionally dependent) with C and U in the selected sample. Note, conditioning on a descendant of X is not the same as directly conditioning on X (such as in the regression of Y on X). Because we are not directly conditioning on X, pathways between Z and Y which include X as a partial mediator are not closed by selection.
Additional note: Selection mechanisms $X + C$ and $X + Y$ have additional pathways between Z and Y via S which can result in bias. Selection on $X + C$ opens the pathway $Z \rightarrow X \rightarrow \boxed{S} \leftarrow C \rightarrow Y$, which again leads to confounding of the $Y - Z$ association by C in the selected sample. And, selection on $X + Y$ opens pathway $Z \rightarrow X \rightarrow \boxed{S} \leftarrow Y$, such that, in the selected sample there is always an association between X and Y even when X does not truly cause Y (i.e., the true causal exposure effect is null); thus, $\hat{\beta}^{2SLS}_{X}$ is biased by selection. Note, because pathway $Z \rightarrow X \rightarrow \boxed{S} \leftarrow Y$ is not via C nor U this latter bias will persist even when the $X - Y$ association is not confounded by C nor U (i.e., $\hat{\beta}^{2SLS}_{X}$ will be biased even in the absence of measured and unmeasured confounders).

Selection depending on Y only

Selection depending on Y (Figure 1h) has the special property that $\hat{\beta}^{2SLS}_{X}$ is only biased by selection when X causes Y (i.e., the true causal exposure effect is not null).
Explanation: When X causes Y, conditioning on S unblocks pathways $Z \rightarrow X \rightarrow Y \leftarrow U$ and $Z \rightarrow X \rightarrow Y \leftarrow C$ because S is a descendant of collider Y; thus, selection induces an association between Z and C, and between Z and U in the selected sample. In addition, X is also a descendant of S via Y (i.e. $X \rightarrow Y \rightarrow S$) and so conditioning on S unblocks pathways which include X as a collider (e.g., $Z \rightarrow X \leftarrow C$). When X does not cause

Y, Z is not associated with C nor U in the selected sample because pathways $Z \rightarrow X \quad\quad Y \leftarrow U$ and $Z \rightarrow X \quad\quad Y \leftarrow U$ are now blocked, and S is no longer a descendant of X (i.e., $X \quad\quad Y \rightarrow S$). Therefore, when X does not cause Y the $Y - Z$ association remains unconfounded in the selected sample, and $\hat{\beta}_X^{2SLS}$ is not biased by selection.

<u>Selection depending on Y and Z</u>

When selection depends on $Y + Z$ (Figure 1i) $\hat{\beta}_X^{2SLS}$ is biased by selection because the instrument is directly associated with the outcome (i.e., the $Y - Z$ association is not via the exposure, X), which is a violation of another IV assumption.

Explanation: Selection unblocks a pathway between Z and Y which does not include X, (i.e., $Z \rightarrow \boxed{\text{S}} \leftarrow Y$) such that in the selected sample Z is directly associated with Y. Of course since this pathway does not include $X \rightarrow Y$ then selection results in bias regardless of whether or not X causes Y. Furthermore, because pathway $Z \rightarrow \boxed{\text{S}} \leftarrow Y$ is not via C nor U then $\hat{\beta}_X^{2SLS}$ will be biased even when the $X - Y$ association is not confounded by C nor U.

Additional note: When X causes Y, selection also results in confounding of the $Y - Z$ association by C and U. As discussed for selection mechanism Y, provided X causes Y, selection unblocks pathways in which Y and X are colliders; thus, inducing an association between Z and C, and Z and U in the selected sample. In practice, selection may not depend directly on Y or Z. For example, selection may depend on Z and unmeasured factors V, where V affects outcome Y (see eFigure 1a; a similar example shown in Boef et al [3]). The consequences of selection on $V + Z$ would be similar to selection on $Y + Z$, violating the same two IV assumptions.

<u>Exposure effect conditional on C</u>

For selection mechanisms X, $X + C$, $X + Y$, Y, and $Y + Z$ estimating the causal exposure effect conditional on C, $\hat{\beta}_{X|C}^{2SLS}$, reduces the level of selection bias because conditioning on C eliminates confounding by C (i.e., blocks all pathways between Z and Y via C). However, $\hat{\beta}_{X|C}^{2SLS}$ remains biased by selection because the $Y - Z$ association is still confounded by U in the selected sample.

<u>Remaining selection mechanisms for our IV example</u>

We have discussed 10 out of a possible 32 selection mechanisms for our IV analysis example, and if we treat C and U as interchangeable, ignoring that it is only possible to condition on measured confounders C, then this reduces to

16 possible mechanisms. eFigures 1b to 1g depict the DAGs for the 6 selection mechanisms not discussed above, and we can explain when selection does and does not bias $\hat{\beta}_X^{2SLS}$ using one of, or a combination of, the explained selection mechanisms described above (e.g., the explanation for selection mechanism Y applies to selection mechanism $Y + C$).

Detailed description of the simulation study

For our IV analysis example, we investigated the effects of 9 selection mechanisms on the two-stage least squares estimate of the causal exposure effect.

Methods

We ensured the three IV assumptions held true in the full sample. The simulated data were generated under the following model:

$$\begin{gathered} z_i \sim N(0,1),\ c_i \sim N(0,1),\ u_i \sim N(0,1), \\ \epsilon_{xi} \sim N(0,1),\ \epsilon_{yi} \sim N(0,1), \\ x_i = \alpha_0 + \alpha_Z z_i + \alpha_{Z^3} z_i^3 + \alpha_C c_i + \alpha_U u_i + \epsilon_{xi}, \\ y_i = \beta_0 + \beta_X x_i + \beta_C c_i + \beta_U u_i + \epsilon_{yi}, \end{gathered} \tag{1}$$

where, y_i, x_i, z_i, c_i and u_i respectively denote realizations of variables Y, X, Z, C and U for participant i, and ϵ_{xi} and ϵ_{yi} are normally distributed errors. The values of the data model parameters are listed in eTable 1. For simplicity, we set the constant terms, α_0 and β_0, in the equations to zero. We considered a causal exposure effect of 1 (i.e., $\beta_X = 1$) and a null causal exposure effect (i.e., $\beta_X = 0$). We set the values of $\alpha_C, \alpha_U, \beta_C$ and β_U such that, in the full sample (i.e., the selected and unselected participants), the ordinary least squares estimate of β_X (i.e., estimated by regressing Y on X) was biased due to confounding and the level of this bias was close to 0.22.

We considered different instrument strengths (i.e., the amount of variation of the exposure explained by the instrument) by setting the value of α_Z. To ensure consistency across all simulation settings, we measured instrument strength using the partial $R^2_{X|Z}$ statistic rather than the F statistic since the latter is subject to large sampling variability, and so is an unreliable measure of instrument strength [4]. Note, we report the population F statistic of our simulations in the results section below. We chose values of α_Z such that, in the full sample, partial $R^2_{X|Z}$ was close to 0.39 for the strong instrument, and 0.045 for the moderate instrument. As a sensitivity analysis, we also considered an instrument strength of partial $R^2_{X|Z}$ close to 0.15. We ensured

that all instruments had sufficient strength in order to avoid weak instrument bias (i.e., bias due to finite sample size) in the full and selected samples.

We investigated whether the effects of selection differed between a linear ($\alpha_{Z^3} = 0$) and a nonlinear ($\alpha_{Z^3} = 1$) $X - Z$ association. For each combination of true value of exposure effect, form of the $X - Z$ association, and instrument strength we generated $3,000$ simulated datasets, each with $20,000$ participants for the full sample.

Following generation of the full sample datasets, participants were independently selected using the following selection model

$$Pr(\text{participant } i \text{ selected}) \\ = \text{expit}\{\eta_0 + \eta_Z z_i + \eta_C c_i + \eta_X x_i + \eta_Y y_i\},$$

where $\text{expit}\{w\} = \dfrac{\exp\{w\}}{1 + \exp\{w\}}$. Different selection mechanisms were created by setting certain parameters to 0; for example, when selection depended on Z, parameters η_C, η_X and η_Y were set to 0. To ensure a consistent strength of selection, values for the selection model's parameters were chosen such that the mean and standard deviation of the selection probabilities (over the full sample) were the same for all selection mechanisms and simulation settings. Close to 60% of the participants were selected; that is, the mean probability of selection was close to 0.6. The standard deviations were close to 0.2 with one exception for selection mechanism "completely at random", where by definition the selection probabilities were the same for all participants and so the standard deviation was 0. eTable 2 shows the values of the selection model's parameters.

We used Stata command *ivregress* to perform two-stage least squares estimation. And, analyzed the simulation results using the Stata command *simsum* [5]. We also conducted a weighted two-stage least squares analysis, using inverse probability weighting (IPW) [6], in which the weights try to make the selected participants a representative sample of the study population [7].

Results

When there was no selection (i.e., all $20,000$ observations were analyzed), $\hat{\beta}_X^{2SLS}$ was unbiased (i.e., estimates do not systematically differ from the true value) and CI coverage was nominal (i.e., close to 95%) for all simulation settings (causal and noncausal exposure effect, linear and nonlinear $X - Z$ association, and strong and moderate instrument).

eTable 3 presents the simulation results of $\hat{\beta}_X^{2SLS}$ for the moderate and strong instruments, when the true exposure effect was 1 ($\beta_X = 1$) and the

$X - Z$ association was linear. The corresponding results for settings $\beta_X = 1$ and nonlinear $X - Z$, $\beta_X = 0$ and linear $X - Z$, and $\beta_X = 0$ and nonlinear $X - Z$ are presented in eTables 4, 5, and 6, respectively.

Impact of instrument strength

For selection mechanisms $Z + C$, $X + Z$ and $Y + Z$ the level of bias increased with decreasing instrument strength: in comparison to the strong instrument (partial $R^2_{X|Z} = 0.39$), the level of bias was 3 to 4 times larger for the moderate instrument (partial $R^2_{X|Z} = 0.045$) (eTable 3), and between 1.6 to 1.8 times larger for instrument strength partial $R^2_{X|Z} = 0.15$ (eTable 7). When selection did not depend on Z, there were only small differences in the level of bias between the instrument strengths.

For all selection mechanisms, decreasing the instrument strength resulted in larger standard errors: in comparison to the strong instrument, the mean standard errors were 3 to 5 times larger for the moderate instrument, and close to double for instrument strength partial $R^2_{X|Z} = 0.15$. These increases in the standard errors were due to weakening of the instrument, as shown in the IV literature (e.g., [8]). The standard errors of $\hat{\beta}_X^{2SLS}$ were neither underestimated nor overestimated (results of the relative error statistic not shown), and thus selection did not affect estimation of the standard errors. Except for selection mechanism $Y + Z$, the larger standard errors (due to weaker instruments) resulted in higher CI coverages.

Incorrectly concluding non-causality due to selection bias

Despite a biased two-stage least squares estimate and poor CI coverage, 100% of the CIs showed evidence of a positive exposure effect for all simulation settings where the true casual effect was 1. This was in part due to the large sample size and relatively large effect size of 1 (i.e., relatively far from 0). When we lowered the effect size to 0.25, and used the moderate instrument strength, a small (1%) to large percentage (76%) of the CIs incorrectly showed evidence of a null exposure effect (i.e., included the null), where the percentage of CIs including the null effect increased with larger selection bias.

Detailed description of the applied example

We now consider an applied IV analysis examining the causal effect of education on the decision to smoke using data from the UK Biobank study [9] where nonrandom selection of participants into the analysis dataset was

suspected. Although this data analysis has a binary exposure, outcome and instrument the IV analysis was conducted using two-stage least squares estimation in the same manner as described for our example of an IV analysis and in our simulation study.

Smoking is still a major avoidable risk factor for ill health and premature mortality worldwide [10]. Higher educational achievement predicts lower levels of smoking uptake and, among those who started smoking, higher levels of quitting smoking [11, 12]. However, we do not know whether this is due to a causal effect of education on the decision to smoke, or if this association is due to unmeasured confounding factors. To counteract the possible effects of unmeasured confounding an IV analysis was conducted where the instrument was a policy reform, introduced on 1st September 1972, which raised the school leaving age from 15 to 16 years. Individuals who turned 15 before this date could leave school at age 15 whilst younger individuals, who turned 15 after 1st September 1972, had to remain in school at least until age 16. This example uses the policy reform (often referred to as ROSLA, Raising of School Leaving Age) as an instrument for time spent in education. The ROSLA has been used as an instrument in previous examples [13, 14, 15] and for the purposes of this example we assume that the instrument is valid.

The UK Biobank study is a sample of $502,644$ individuals who attended 23 study clinics across the UK, and did not subsequently withdraw from the study, enrolled between 2006 and 2010 [9]. The study achieved a response rate of 5.5% after inviting 9.2 million individuals to participate in the study [16]. The participants of the cohort were relatively young and so participation (i.e., "selection into the study") was unlikely to have been affected by smoking-related mortality. Higher levels of educational achievement predicted attendance at a study clinic, and hence sampling by UK Biobank; for example, quoting from Davies et al [13], "The UK Biobank is a volunteer sample and, as a result, people who left school at 16 years of age were less likely to attend the clinics than previous studies (17.5% versus 33% reported in Clark and Royer [14])". Therefore, it is plausible that study participants were nonrandomly selected depending on the exposure of interest, educational attainment. As discussed earlier in the section on when selection leads to bias, and shown by the simulation study, selection depending on the exposure can bias an IV analysis.

To maximise the plausibility of the IV assumptions, we restricted our analysis to participants who turned 15 within the period of one year before to one year after the introduction of the ROSLA policy. Note, this example is intended to demonstrate the impact of sample selection on linear regression and IV analyses, and not as a comprehensive analysis of the effect of education on smoking in adult life. We further restricted our sample to those

participants born in England or Wales because the ROSLA only affected England and Wales. Among those born in England or Wales, we only included participants who answered questions about the age at which they left school or their highest level of qualification. For the purposes of this example we have assumed that there were no systematic differences between the UK Biobank participants selected for analysis (i.e., aged 15 within ± 1 year of the policy introduction, born in England or Wales and had information on the exposure) and those UK Biobank participants not selected for analysis (i.e., not aged 15 within ± 1 year of the policy introduction, born outside of England and Wales, or no information on the exposure) with respect to the exposure, outcome, or any confounders of the exposure-outcome association. Under this assumption, we have ignored our selection on age at time of the policy introduction, place of birth and whether information on the exposure was observed or missing.

Using the same notation as previously, the outcome Y was a binary variable, equal to one if the participant had ever smoked (i.e., includes ex-smokers and current smokers), and equal to zero if the participant had never smoked. We also considered a second outcome which was also a binary variable, equal to one if, at the time of the study clinic, the participant was a current smoker, and equal to zero if the participant did not smoke at that time (i.e., included ex-smokers and never smokers). The distributions of data on the instrument, exposure, and these two outcomes are shown in eTable 14. Note, a small number of individuals left school before the age of 16 after the reform had been introduced. Excluding these individuals gave similar results to those reported in table 2 of the main paper (results not shown). Separate analyses were performed on each outcome using the same exposure and instrument. The exposure X was a binary variable, equal to one if the participant had left school age 16 or older, and equal to zero otherwise. The instrument, Z, was also a binary variable, equal to one if the participant turned 15 after the policy reform was introduced, and equal to zero otherwise. There were a few measured confounders, C, of the exposure-outcome association (e.g., sex, month of birth) but we suspect there were many unmeasured confounders, U. Although the validity of the instrument did not require conditioning on any measured variables, following Clark and Royer [14] we have adjusted for sex and month of birth.

We conducted the IV analysis using the linear probability model. This model is a form of two-stage least squares estimation in which the outcome, exposure and instrument are binary, and the causal exposure effect is on the risk difference scale [17]. We note, fitting a linear regression model when the dependent variable is binary may produce predicted values outside of the 0 to 1 range [18]. Robust standard errors were calculated to account for

assumptions about homogeneous exposure effects and the outcome distributions. For comparison, we also considered the equivalent standard analysis; that is, the linear regression of Y on X (e.g., current smoking on "left school age 16 or older"), also with robust standard errors. Although the standard analysis may be biased by unmeasured confounding of the exposure-outcome association, we know from the missing data literature (e.g., [19]) that the results of the standard analysis are not biased by selection on the exposure, X.

A weighted IV analysis, in which the weights try to make the selected participants a representative sample of the study population, (e.g., [7]) can account for unmeasured confounding and selection bias. We used the weighting method known as inverse probability weighting [6]. Under the assumption that selection only depended on the exposure X, the weights were the inverse of the conditional probabilities of selection given the exposure. (See the next section regarding calculation of the weights). The selected participants who left school age 15 were weighted by 34.29, and the selected participants who left school age 16 or older were weighted by 16.36 (weights rounded to 2 decimal places). So, those participants suspected to be under-represented in the selected sample (i.e., left school age 15) contributed more to the analysis than those suspected to be over-represented in the selected sample (i.e. left school age 16 or older). For comparison, we carried out a weighted linear regression analysis using the same weights. Information on calculation of the weights is given below. For both weighted analyses the uncertainty of the weights was taken into account using the sandwich variance estimator [20], although the standard errors of the weights were small.

Calculation of the weights

To calculate the weights we have made three key assumptions: (1) that the exposure (an extra year of compulsory education) was the only factor causing selection into UK Biobank; (2) that there was no effect of cigarette smoking on selection into UK Biobank (although Fry et al, [16], would suggest otherwise); and (3) that the probability of selection for our restricted sample is the same as the probability of selection for all invited participants to the UK Biobank study. This example is for illustrative purposes only; in practice a thorough investigation of the factors likely to affect selection would be necessary.

Under the assumption that selection S only depended on the exposure X, the weights were the inverse of the conditional probabilities of selection given the exposure, $Pr(S = 1|X)$. We can see from figure 1e that under this selection mechanism, selection is independent of outcome Y and instrument

Z after conditioning on X (i.e., pathways between S and Z, and S and Y are closed after conditioning on X). To calculate $Pr(S = 1|X)$ we use Bayes theorem

$$Pr(S|X) = \frac{Pr(X|S)Pr(S)}{Pr(X)}. \tag{2}$$

First, let us calculate the conditional probability of selection for those participants who left school at age 15 (i.e., $Pr(S = 1|X = 0)$). We used information from the 2011 UK census [21] to approximate information on all individuals invited to participate in the study (i.e., the selected and unselected). Prior to the introduction of the policy reform (i.e., $Z = 0$), September 1972, 33% of individuals left school at age 15, and so we set $Pr(X = 0|Z = 0) = 0.33$. Among the selected participants, 17.5% left school at age 15 prior to September 1972; that is, $Pr(X = 0|S = 1, Z = 0) = 0.175$. After the introduction of the policy reform, a small number of individuals did leave school at age 15. However, for the purposes of our illustrative example we have assumed from September 1972 onwards that all individuals left school aged 16 or older; that is, $Pr(X = 0|Z = 1) = 0$ and $Pr(X = 0|S = 1, Z = 1) = 0$. Therefore, $Pr(X = 0) = Pr(X = 0|Z = 0)Pr(Z = 0)$ and $Pr(X = 0|S = 1) = Pr(X = 0|S = 1, Z = 0)Pr(Z = 0)$. For the probability of selection, we know that $502,644$ out of 9.2 million invited participants attended the study clinic, and so $Pr(S = 1) = 502644/9200000 = 0.055$ (2 decimal places). Putting everything together we have:

$$\begin{aligned} Pr(S = 1|X = 0) &= \frac{Pr(X = 0|S = 1)Pr(S = 1)}{Pr(X = 0)} \\ &= \frac{Pr(X = 0|S = 1, Z = 0)Pr(Z = 0)Pr(S = 1)}{Pr(X = 0|Z = 0)Pr(Z = 0)} \\ &= \frac{0.175 \times 0.055}{0.33} \\ &= 0.02916667. \end{aligned} \tag{3}$$

Similarly, we can calculate the conditional probability of selection for those participants who left school age 16 or older (i.e., $Pr(S = 1|X = 1)$). From the UK census we have $Pr(X = 1|Z = 0) = 1 - 0.33$, and from the selected sample we have $Pr(X = 1|S = 1, Z = 0) = 1 - 0.175$. Given our assumption that post September 1972 all individuals left school aged 16 or older, then $Pr(X = 1|Z = 1) = 1$ and $Pr(X = 1|S = 1, Z = 1) = 1$. Since individuals could leave school age 16 or older before and after September 1972 then $Pr(X = 1) = 0.67 \times Pr(Z = 0) + Pr(Z = 1) = 1 - 0.33 \times Pr(Z = 0)$, and $Pr(X = 1|S = 1) = 0.825 \times Pr(Z = 0) + Pr(Z = 1) = 1 - 0.175 \times Pr(Z =$

0). The probability $Pr(Z = 0)$ is the proportion of the 9.2 million invited participants to which the introduction of the policy reform did not apply; that is, the proportion that turned 15 before September 1972. The recruitment period for the UK Biobank study was between 2006 and 2010, and so the mid-point was 2008. Participants unaffected by the policy reform had to have turned 51 years before September 2008. We approximate $Pr(Z = 0)$ using population estimates for England and Wales, Scotland and Northern Ireland supplied by the Office for National Statistics. Based on these population estimates for June 2008, among those aged between 40 to 70 years (UK Biobank's sampling ages) the proportion over 50 was 0.58. So, we estimated that 58% of the invited UK Biobank participants had turned 51 years before September 2008 (i.e., $Pr(Z = 0) = 0.58$). Putting everything together we have:

$$\begin{aligned} Pr(S=1|X=1) &= \frac{Pr(X=1|S=1)Pr(S=1)}{Pr(X=1)} \\ &= \frac{[1-0.175 \times Pr(Z=0)]Pr(S=1)}{1-0.33 \times Pr(Z=0)} \\ &= \frac{[1-0.175 \times 0.58] \times 0.055}{1-0.33 \times 0.58} \\ &= 0.06111489. \end{aligned} \tag{4}$$

Therefore, the selected participants who left school age 15 were weighted by 34.29 and the selected participants who left school age 16 or older were weighted by 16.36 (weights rounded to 2 decimal places).

Further comments

This applied example was for illustrative purposes only and so our analysis has some limitations.

In practice a thorough investigation of the factors likely to affect selection would be necessary. Fry et al [16] compared various sociodemographic, physical, lifestyle and health-related characteristics of the cohort to data sources representative of the intended study population; thus, allowing the authors to compare the participants (the selected sample) to the nonparticipating invitees (the unselected sample). Compared with the general UK population, UK Biobank participants were more highly educated, older, female, live in less socioeconomically deprived areas, and less likely to be obese, to smoke and to drink alcohol daily, and had fewer self-reported adverse health outcomes [13, 16]. Consequently, for our analysis, selection may not only depend

on the exposure but also on the outcome and unmeasured confounders (e.g., obesity). Because our weighted analysis only allowed for selection on the exposure it may only partially account for the selection bias [22].

We do not directly observe the unselected sample and instead calculate the weights using external nationally representative data sources. Therefore, our weights for selection on the exposure were only approximations. Given the large sample size of UK Biobank moderate changes in the weights had a small impact on the results of the weighted IV analysis; for example, for outcome current smoker, a weighted IV analysis using weights 100 and 12 (for participants who left school at age 15 and ≥ 16, respectively) gave a risk difference of -7.29% [95% confidence interval $-9.64\%, -4.93\%$]. Typically, an analyst does not have access to the individual level data of these external data sources and instead relies on reported results. Generating (approximately) correct weights based on multiple factors (e.g., level of education, smoking status, sex, obesity) using these external data sources is difficult because they usually do not report the required conditional probabilities. Therefore, the analyst must consider the pros and cons of calculating approximately correct weights versus including all potential selection factors. In all cases, a weighted analysis using external data sources should be considered as a sensitivity analysis and not as the main analysis. Further work is needed on how to conduct IPW using external data.

IPW [23] and multiple imputation (MI) [24] have been used to appropriately account for selection bias in an IV analysis. MI is generally more efficient because it uses all observed data on the analysis variables, whereas IPW discards any observed data observed on the unselected sample. For our analysis, IPW was an appropriate choice because there was no available data on the unselected.

As noted above, when selection depends on unmeasured data then any MI or IPW analysis must be part of a sensitivity analysis. Sensitivity analyses have been proposed to account for nonrandom selection in an IV analysis (e.g., recovery of the local average treatment effect when selecting on treatment received [7], IV analyses with nonignorable missing covariates [24, 25]). However, the proposed methods are specific to a particular analysis, and may not be accessible to non-technical analysts without the provision of appropriate guidelines and easy to use software (although the authors did supply their R code to implement their analyses). More general, user friendly methods may be found in the missing data literature (e.g., NARFCS multiple imputation command [26]). Further research is needed to provide IV analysts with easy to use methods, software and guidelines on how to conduct sensitivity analyses when selection depends on unmeasured data.

Simulation study based on the applied example

Based on our applied example, we conducted a simulation study where the outcome Y, exposure X and instrument Z were binary variables, and continuous unmeasured confounder U. We investigated the effects of 9 selection mechanisms on the two-stage least squares estimate of the causal exposure effect, estimated using the linear probability model. The simulated data were generated under the following model:

$$\begin{aligned} z_i &\sim Bernoulli(0.492),\ u_i \sim N(0,1), \\ x_i &\sim Bernoulli(\text{expit}\{0.802 + 1.30z_i - 0.740u_i\}), \\ y_i &\sim Bernoulli(\text{expit}\{-0.586 - 0.200x_i + 0.740u_i\}), \end{aligned} \tag{5}$$

where, y_i, x_i, z_i and u_i respectively denote realizations of variables Y, X, Z and U for participant i. Where possible, the values of the data model parameters were based on the applied example. We set the instrument strength, in the full sample, as partial $R^2_{X|Z}$ close to 0.056. The true causal exposure effect was set to -0.045, and we set the level of confounding such that, in the full sample, the ordinary least squares estimate of the causal exposure effect was close to -14.1. We generated $3,000$ simulated datasets, each with $20,000$ participants for the full sample.

Similarly to our simulation study based on all continuous variables, participants were independently selected using the following selection model

$$\begin{aligned} &Pr(\text{participant } i \text{ selected}) \\ &\quad = \text{expit}\{\eta_0 + \eta_Z z_i + \eta_U u_i + \eta_X x_i + \eta_Y y_i\}. \end{aligned}$$

For each selection mechanism, close to 60% of the participants were selected and the standard deviation of the selection probabilities was close to 0.2 (except for selection completely at random). eTable 15 shows the values of the selection model's parameters.

We used Stata command *ivregress* to perform two-stage least squares estimation, and analyzed the simulation results using the Stata command *simsum* [5]. We also conducted a weighted two-stage least squares analysis, using IPW, to correct for nonrandom selection.

eTable 16 shows the results of the (unweighted) two-stage least squares analyses. When there was no selection (full sample), selection was completely at random or depended on Z only $\hat{\beta}_X^{2SLS}$ was unbiased and CI coverage was nominal. For selection mechanisms X and $X+Z$ $\hat{\beta}_X^{2SLS}$ was positively biased,

and for the remaining selection mechanisms $\hat{\beta}_X^{2SLS}$ was negatively biased. The direction of the bias depended on the direction of the induced $Z-U$ association (n.b., the true $Y-U$ association was positive and the true $Y-Z$ association was negative). For example, when selection depended on X the $Z-U$ association was positive. Therefore, U was a negative confounder of the $Y-Z$ association [27]; that is, the unadjusted estimate of Y given Z was pushed closer towards the null than the truth, leading to positive bias of coefficient $\hat{E}(Y|Z)$ and so positive bias of $\hat{\beta}_X^{2SLS}$. Whereas, when selection depended on $Y+Z$ the $Z-U$ association was negative. Therefore, U was a positive confounder of the $Y-Z$ association; that is, the unadjusted estimate of Y given Z was pulled further away from the null than the truth, leading to negative bias of coefficient $\hat{E}(Y|Z)$ and so negative bias of $\hat{\beta}_X^{2SLS}$.

For selection mechanisms $X+C$ and Y CI coverage was nominal because the magnitude of the bias was relatively small and, due to the moderate strength of the instrument, the standard errors were large enough to allow for the small amount of bias. For the remaining selection mechanisms that resulted in bias, CI undercoverage was poor (78%) to severe (0%).

As expected, the weighted two-stage least squares analyses gave unbiased estimates of $\hat{\beta}_X^{2SLS}$ with nominal CI coverages for all selection mechanisms (eTable 17).

eAppendix references

[1] MA Hernán, S Hernández-Diaz, and JM Robins. A structural approach to selection bias. *Epidemiology*, 15:615–625, 2004.

[2] S Greenland. Quantifying biases in causal models: classical confounding vs collider-stratification bias. *Epidemiology*, 14:300–306, 2003.

[3] AGC Boef, S le Cessie, and OM Dekkers. Mendelian randomization studies in the elderly. *Epidemiology*, 26(2):e15–e16, 2015.

[4] S Burgess and SG Thompson. *Mendelian randomization. methods for using genetic variants in causal estimation.* Boca Raton: Chapman & Hall/CRC, 2015.

[5] IR White. simsum: Analyses of simulation studies including monte carlo error. *Stata J*, 10:369–385, 2010.

[6] SR Seaman and IR White. Review of inverse probability weighting for dealing with missing data. *Stat Methods Med Res*, 22:278–295, 2013.

[7] A Ertefaie, D Small, J Flory, and S Hennessy. Selection bias when using instrumental variable methods to compare two treatments but more than two treatments are available. *Int J Biostat*, 12:219–232, 2016.

[8] M Baiocchi, J Cheng, and DS Small. Instrumental variable methods for causal inference. *Stat Med*, 33:2297–2340, 2014.

[9] C Sudlow, J Gallacher, N Allen, V Beral, P Burton, J Danesh, and P Downey. Uk biobank: an open access resource for identifying the causes of a wide range of complex diseases of middle and old age. *PLoS Med*, 12(3):e1001779, 2015. doi: 10.1371/journal.pmed.1001779.

[10] P Jha. Avoidable global cancer deaths and total deaths from smoking. *Nat Rev Cancer*, 9:655–664, 2009.

[11] SH Gage, J Bowden, G Davey Smith, and MR Munafò. Investigating causality in associations between education and smoking: A two-sample mendelian randomization study. *Int J Epidemiol*, 47:1131–1140, 2018.

[12] BJ Jeffris, C Power, H Graham, and O Manor. Changing social gradients in cigarette smoking and cessation over two decades of adult follow-up in a british birth cohort. *J Public Health*, 26:13–18, 2004.

[13] NM Davies, M Dickson, G Davey Smith, G van den Berg, and F Windmeijer. The causal effects of education on health outcomes in the uk biobank. *Nat Hum Behav*, 2018. URL `https://doi.org/10.1038/s41562-017-0279-y`.

[14] D Clark and H Royer. The effect of education on adult mortality and health: Evidence from britain. *Am Econ Rev*, 103:2087–2120, 2013.

[15] C Harmon and I Walker. Estimates of the economic return to schooling for the united kingdom. *Am Econ Rev*, 85:1278–1286, 1995.

[16] A Fry, TJ Littlejohns, C Sudlow, N Doherty, L Adamska, T Sprosen, R Collins, and NE Allen. Comparison of sociodemographic and health-related characteristics of uk biobank participants with those of the general population. *Am J Epidemiol*, 186:1026–1034, 2017. doi: 10.1093/aje/kwx246.

[17] OH Klungel, M Jamal Uddin, A de Boer, SV Belitser, RH Groenwold, and KC Roes. Instrumental variable analysis in epidemiologic studies: An overview of the estimation methods. *Pharm Anal Acta*, 353, 2015. doi: 10.4172/2153-2435.1000353.

[18] AC Cameron and PK Trivedi. *Microeconometrics: methods and applications*. Cambridge: Cambridge University Press, 2005.

[19] JW Bartlett, O Harel, and JR Carpenter. Asymptotically unbiased estimation of exposure odds ratios in complete records logistic regression. *Am J Epidemiol*, 182:730–736, 2015.

[20] J Robins, A Rotnitzky, and L Zhao. Estimation of regression coefficients when some regressors are not always observed. *J Am Stat Assoc*, 89: 846866, 1994.

[21] Office for National Statistics. Census statistics for the population of england and wales, 2011. URL `https://www.ons.gov.uk/census/2011census`.

[22] S Seaman and I White. Inverse probability weighting with missing predictors of treatment assignment or missingness. *Commun Stat Theory Methods*, 43:3499–3515, 2014.

[23] C Canan, C Lesko, and B Lau. Instrumental variable analyses and selection bias. *Epidemiology*, 28:396–398, 2017.

[24] F Yang, SA Lorch, and DS Small. Estimation of causal effects using instrumental variables with nonignorable missing covariates: application to effect of type of delivery nicu on premature infants. *Ann Appl Stat*, 8:48–73, 2014.

[25] A Ertefaie, JH Flory, S Hennessy, and DS Small. Instrumental variable methods for continuous outcomes that accommodate nonignorable missing baseline values. *Am J Epidemiol*, 185:1233–1239, 2017.

[26] F Tompsett, DM amd Leacy, M Moreno-Betancur, J. Heron, and IR White. On the use of the not-at-random fully conditional specification (narfcs) procedure in practice. *Stat Med*, 37:23382353, 2018.

[27] A Mehio-Sibai, M Feinleib, TA Sibai, and HK Armenian. A positive or a negative confounding variable? a simple teaching aid for clinicians and students. *Ann Epidemiol*, 15(6):421–423, 2005.

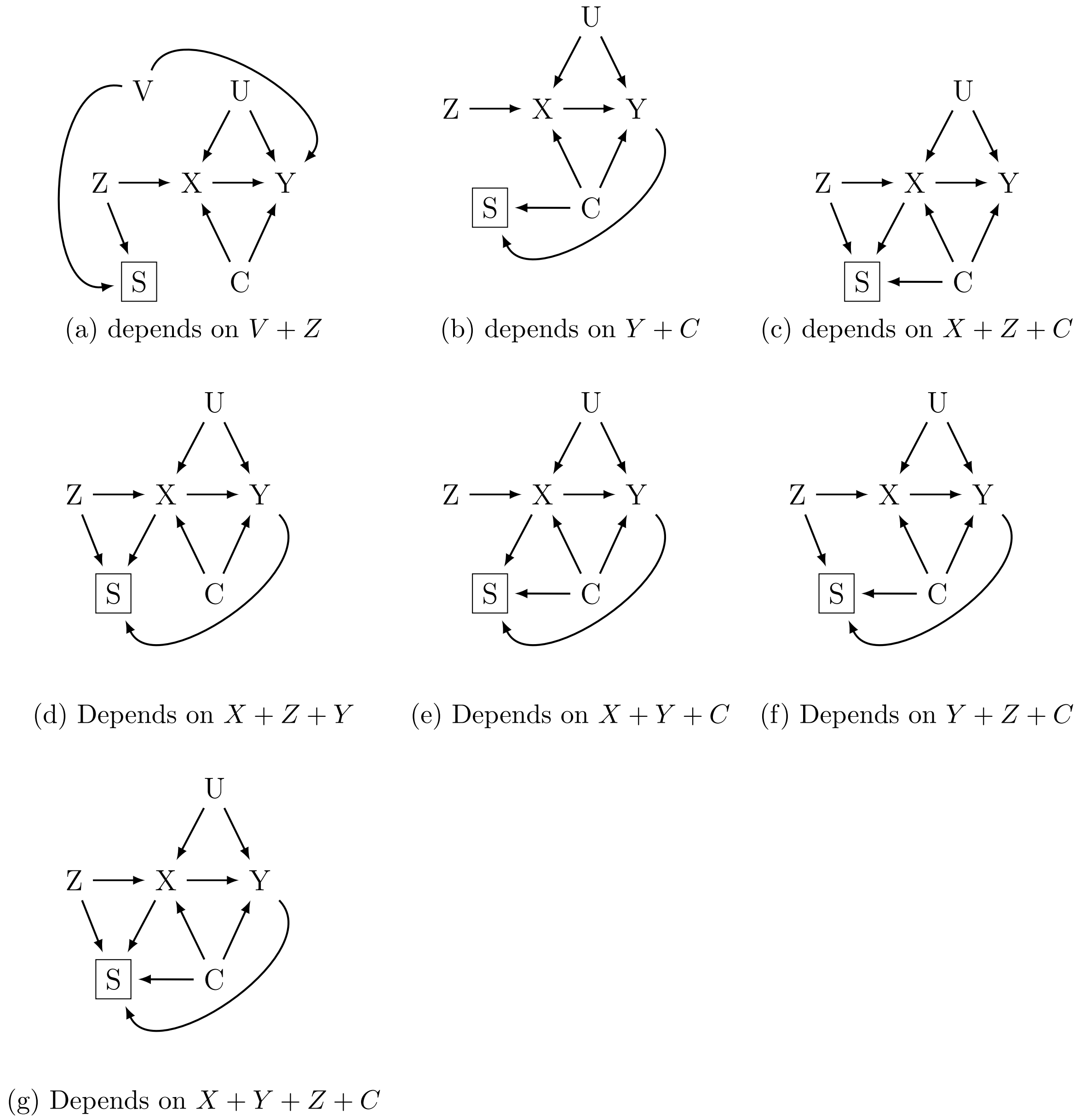

(a) depends on $V + Z$

(b) depends on $Y + C$

(c) depends on $X + Z + C$

(d) Depends on $X + Z + Y$

(e) Depends on $X + Y + C$

(f) Depends on $Y + Z + C$

(g) Depends on $X + Y + Z + C$

eFigure 1: Directed acyclic graphs of an instrumental variable analysis under seven additional selection mechanisms.

eTable 1: Values of the data model's parameters according to whether the exposure−instrument ($X - Z$) association was linear or nonlinear, instrument strength was moderate or strong, and true causal exposure effect was 1 or 0. For all simulation settings, constant coefficients $\alpha_0 = \beta_0 = 0$, and confounders' coefficients $\alpha_C = \beta_U = \beta_C = \alpha_U$.

$X - Z$ association	Instrument Strength	Causal exposure effect	α_Z	α_Z^3	α_U
Linear	Moderate	0 or 1	0.248	0	0.380
Linear	Strong	0 or 1	1.00	0	0.531
Nonlinear	Moderate	0	−2.34	1	1.04
Nonlinear	Moderate	1	−2.35	1	1.04
Nonlinear	Strong	0 or 1	−0.320	1	1.43

eTable 2: Values of the selection model's parameters for 9 selection mechanisms, including selection completely at random (SCAR), according to whether the exposure−instrument ($X - Z$) association was linear or nonlinear, instrument strength was moderate or strong, and true value of exposure effect (β_X) was 1 or 0.

Selection is/ depends on	$X - Z$ association	Instrument	β_X	η_0	η_Z	η_C	η_X	η_Y
SCAR	Linear/nonlinear	Moderate/strong	0/1	0.425	0	0	0	0
Z	Linear/nonlinear	Moderate/strong	0/1	0.490	1.00	0	0	0
$Z + C$	Linear/nonlinear	Moderate/strong	0/1	0.500	0.700	0.700	0	0
X	Linear	Moderate	0/1	0.517	0	0	0.875	0
	Linear	Strong	0/1	0.517	0	0	0.640	0
	Nonlinear	Moderate	0	0.510	0	0	0.435	0
	Nonlinear	Moderate	1	0.500	0	0	0.425	0
	Nonlinear	Strong	0	0.500	0	0	0.325	0
	Nonlinear	Strong	1	0.490	0	0	0.320	0
$X + C$	Linear	Moderate	0/1	0.500	0	0.575	0.575	0
	Linear	Strong	0/1	0.500	0	0.475	0.475	0
	Nonlinear	Moderate	0	0.500	0	0.340	0.340	0
	Nonlinear	Moderate	1	0.500	0	0.340	0.340	0
	Nonlinear	Strong	0	0.500	0	0.260	0.260	0
	Nonlinear	Strong	1	0.500	0	0.265	0.265	0
$X + Z$	Linear	Moderate	0/1	0.500	0.600	0	0.600	0
	Linear	Strong	0/1	0.500	0.425	0	0.425	0
	Nonlinear	Moderate	0	0.500	0.425	0	0.425	0
	Nonlinear	Strong	0	0.500	0.270	0	0.270	0
	Nonlinear	Moderate	1	0.500	0.425	0	0.425	0
	Nonlinear	Strong	1	0.500	0.275	0	0.275	0
Y	Linear	Moderate	0	0.500	0	0	0	0.880
	Linear	Moderate	1	0.500	0	0	0	0.550
	Linear	Strong	0	0.500	0	0	0	0.800
	Linear	Strong	1	0.500	0	0	0	0.435
	Nonlinear	Moderate	0	0.500	0	0	0	0.570
	Nonlinear	Moderate	1	0.500	0	0	0	0.270
	Nonlinear	Strong	0	0.500	0	0	0	0.450
	Nonlinear	Strong	1	0.500	0	0	0	0.200
$X + Y$	Linear	Moderate	0	0.500	0	0	0.550	0.550
	Linear	Moderate	1	0.500	0	0	0.355	0.355
	Linear	Strong	0	0.500	0	0	0.440	0.440
	Linear	Strong	1	0.500	0	0	0.265	0.265
	Nonlinear	Moderate	0	0.505	0	0	0.275	0.275
	Nonlinear	Moderate	1	0.500	0	0	0.170	0.170
	Nonlinear	Strong	0	0.500	0	0	0.200	0.200
	Nonlinear	Strong	1	0.500	0	0	0.122	0.122
$Y + Z$	Linear	Moderate	0	0.500	0.650	0	0	0.650
	Linear	Moderate	1	0.500	0.460	0	0	0.460
	Linear	Strong	0	0.500	0.625	0	0	0.625
	Linear	Strong	1	0.500	0.350	0	0	0.350
	Nonlinear	Moderate	0	0.500	0.490	0	0	0.490
	Nonlinear	Moderate	1	0.500	0.265	0	0	0.265
	Nonlinear	Strong	0	0.500	0.410	0	0	0.410
	Nonlinear	Strong	1	0.500	0.185	0	0	0.185

eTable 3: Summary of the multivariate normal simulation results of the two-stage least squares estimate of the casual exposure effect, $\hat{\beta}_X^{2SLS}$, for true value of 1 and linear $X - Z$ association: Mean (standard deviation) of the partial $R^2_{X|Z}$ of the $X - Z$ association, bias of $\hat{\beta}_X^{2SLS}$, mean of the estimated standard errors, SE, of $\hat{\beta}_X^{2SLS}$, and coverage percentage of the 95% confidence interval for $\hat{\beta}_X^{2SLS}$. Monte Carlo errors in brackets, except for partial $R^2_{X|Z}$.

Full sample/	Moderate Instrument				Strong Instrument			
Selection	partial $R^2_{X\|Z}$	Bias	Mean SE	Coverage	partial $R^2_{X\|Z}$	Bias	Mean SE	Coverage
Full sample	0.0452<br>(0.00285)	0.000499<br>(0.000590)	0.0326<br>(0.0000213)	95.4<br>(0.384)	0.390<br>(0.00535)	0.000181<br>(0.000160)	0.00884<br>(0.00000201)	95.3<br>(0.385)
Completely at random	0.0452<br>(0.00370)	0.000840<br>(0.000758)	0.0420<br>(0.0000360)	95.4<br>(0.382)	0.390<br>(0.00695)	0.000286<br>(0.000205)	0.0114<br>(0.00000341)	95.3<br>(0.385)
on Z	0.0384<br>(0.00338)	0.000144<br>(0.000832)	0.0460<br>(0.0000419)	95.2<br>(0.390)	0.350<br>(0.00693)	0.000121<br>(0.000226)	0.0125<br>(0.00000393)	94.8<br>(0.404)
on Z + C	0.0322<br>(0.00317)	−0.153<br>(0.000951)	0.0525<br>(0.0000591)	15.2<br>(0.655)	0.353<br>(0.00698)	−0.0470<br>(0.000227)	0.0125<br>(0.00000406)	3.33<br>(0.328)
on X	0.0382<br>(0.00345)	−0.0423<br>(0.000915)	0.0502<br>(0.0000483)	87.4<br>(0.606)	0.348<br>(0.00691)	−0.0429<br>(0.000243)	0.0133<br>(0.00000426)	9.67<br>(0.540)
on X + C	0.0425<br>(0.00364)	−0.0384<br>(0.000854)	0.0461<br>(0.0000422)	87.7<br>(0.600)	0.370<br>(0.00690)	−0.0432<br>(0.000229)	0.0126<br>(0.00000391)	6.90<br>(0.463)
on X + Z	0.0163<br>(0.00229)	−0.159<br>(0.00142)	0.0781<br>(0.000125)	46.1<br>(0.910)	0.325<br>(0.00696)	−0.0404<br>(0.000249)	0.0136<br>(0.00000458)	15.9<br>(0.668)
on Y	0.0425<br>(0.00366)	−0.0833<br>(0.000812)	0.0441<br>(0.0000397)	52.7<br>(0.912)	0.373<br>(0.00693)	−0.0704<br>(0.000223)	0.0121<br>(0.00000374)	0.00<br>(0.00)
on X + Y	0.0406<br>(0.00355)	−0.0857<br>(0.000866)	0.0472<br>(0.0000436)	55.1<br>(0.908)	0.362<br>(0.00692)	−0.0688<br>(0.000231)	0.0127<br>(0.00000398)	0.00<br>(0.00)
on Y + Z	0.0241<br>(0.00275)	−0.399<br>(0.00122)	0.0667<br>(0.0000969)	0.00<br>(0.00)	0.342<br>(0.00699)	−0.0980<br>(0.000240)	0.0131<br>(0.00000429)	0.00<br>(0.00)

eTable 4: Summary of the multivariate normal simulation results of the two-stage least squares estimate of the casual exposure effect, $\hat{\beta}_X^{2SLS}$, for true value of 1, and nonlinear $X - Z$ association: Mean (standard deviation) of the partial $R^2_{X|Z}$ of the $X - Z$ association, bias of $\hat{\beta}_X^{2SLS}$, mean of the estimated standard errors, SE, of $\hat{\beta}_X^{2SLS}$, and coverage percentage of the 95% confidence interval for $\hat{\beta}_X^{2SLS}$. Monte Carlo errors in brackets, except for partial $R^2_{X|Z}$.

Full sample/	Moderate Instrument				Strong Instrument			
Selection	partial $R^2_{X\|Z}$	Bias	Mean SE	Coverage	partial $R^2_{X\|Z}$	Bias	Mean SE	Coverage
Full sample	0.0448	0.000196	0.0193	95.3	0.393	0.000110	0.00596	95.3
	(0.00498)	(0.000348)	(0.0000286)	(0.388)	(0.00663)	(0.000107)	(0.00000254)	(0.386)
Completely at random	0.0449	0.000179	0.0250	95.0	0.393	0.000130	0.00766	95.1
	(0.00652)	(0.000451)	(0.0000486)	(0.398)	(0.00866)	(0.000138)	(0.00000426)	(0.394)
on Z	0.0314	−0.000305	0.0314	95.1	0.350	0.0000716	0.00865	95.1
	(0.00552)	(0.000567)	(0.0000736)	(0.395)	(0.00829)	(0.000157)	(0.00000506)	(0.394)
on Z + C	0.0267	−0.176	0.0388	0.00	0.352	−0.0485	0.00877	0.00
	(0.00520)	(0.000794)	(0.000118)	(0.00)	(0.00840)	(0.000162)	(0.00000529)	(0.00)
on X	0.0330	0.00117	0.0319	94.9	0.339	−0.0519	0.00936	0.00
	(0.00571)	(0.000574)	(0.0000732)	(0.400)	(0.00826)	(0.000173)	(0.00000574)	(0.00)
on X + C	0.0335	−0.00919	0.0315	94.6	0.350	−0.0501	0.00908	0.00
	(0.00569)	(0.000569)	(0.0000709)	(0.414)	(0.00833)	(0.000168)	(0.00000545)	(0.00)
on X + Z	0.0177	−0.132	0.0492	18.4	0.322	−0.0599	0.00974	0.00
	(0.00442)	(0.000959)	(0.000189)	(0.708)	(0.00822)	(0.000181)	(0.00000622)	(0.00)
on Y	0.0343	−0.0195	0.0302	90.7	0.365	−0.0503	0.00862	0.00
	(0.00571)	(0.000555)	(0.0000666)	(0.531)	(0.00840)	(0.000159)	(0.00000499)	(0.00)
on X + Y	0.0334	−0.0107	0.0312	94.5	0.354	−0.0526	0.00894	0.00
	(0.00568)	(0.000569)	(0.0000696)	(0.417)	(0.00836)	(0.000165)	(0.00000531)	(0.00)
on Y + Z	0.0233	−0.142	0.0409	3.50	0.348	−0.0644	0.00904	0.00
	(0.00492)	(0.000797)	(0.000127)	(0.336)	(0.00838)	(0.000169)	(0.00000547)	(0.00)

eTable 5: Summary of the multivariate normal simulation results of the two-stage least squares estimate of the casual exposure effect, $\hat{\beta}_X^{2SLS}$, for true value of 0, and linear $X-Z$ association: Mean (standard deviation) of the partial $R^2_{X|Z}$ of the $X-Z$ association, bias of $\hat{\beta}_X^{2SLS}$, mean of the estimated standard errors, SE, of $\hat{\beta}_X^{2SLS}$, and coverage percentage of the 95% confidence interval for $\hat{\beta}_X^{2SLS}$. Monte Carlo errors in brackets, except for partial $R^2_{X|Z}$.

Full sample/	Moderate Instrument				Strong Instrument			
Selection	partial $R^2_{X\|Z}$	Bias	Mean SE	Coverage	partial $R^2_{X\|Z}$	Bias	Mean SE	Coverage
Full sample	0.0452	0.000499	0.0326	95.4	0.390	0.000181	0.00884	95.3
	(0.00285)	(0.000590)	(0.0000213)	(0.384)	(0.00535)	(0.000160)	(0.00000201)	(0.385)
Completely at random	0.0452	0.000840	0.0420	95.4	0.390	0.000286	0.0114	95.3
	(0.00370)	(0.000758)	(0.0000360)	(0.382)	(0.00695)	(0.000205)	(0.00000341)	(0.385)
on Z	0.0384	0.000144	0.0460	95.2	0.350	0.000121	0.0125	94.8
	(0.00338)	(0.000832)	(0.0000419)	(0.390)	(0.00693)	(0.000226)	(0.00000393)	(0.404)
on Z + C	0.0322	−0.153	0.0525	15.2	0.353	−0.0470	0.0125	3.33
	(0.00317)	(0.000951)	(0.0000591)	(0.655)	(0.00698)	(0.000227)	(0.00000406)	(0.328)
on X	0.0382	−0.0423	0.0502	87.4	0.348	−0.0429	0.0133	9.67
	(0.00345)	(0.000915)	(0.0000483)	(0.606)	(0.00691)	(0.000243)	(0.00000426)	(0.540)
on X + C	0.0425	−0.0384	0.0461	87.7	0.370	−0.0432	0.0126	6.90
	(0.00364)	(0.000854)	(0.0000422)	(0.600)	(0.00690)	(0.000229)	(0.00000391)	(0.463)
on X + Z	0.0163	−0.159	0.0781	46.1	0.325	−0.0404	0.0136	15.9
	(0.00229)	(0.00142)	(0.000125)	(0.910)	(0.00696)	(0.000249)	(0.00000458)	(0.668)
on Y	0.0457	0.00108	0.0385	95.6	0.395	0.000284	0.0105	95.7
	(0.00370)	(0.000693)	(0.0000323)	(0.373)	(0.00695)	(0.000189)	(0.00000309)	(0.369)
on X + Y	0.0425	−0.0833	0.0441	52.7	0.373	−0.0716	0.0121	0.00
	(0.00366)	(0.000812)	(0.0000397)	(0.912)	(0.00693)	(0.000224)	(0.00000374)	(0.00)
on Y + Z	0.0357	−0.417	0.0535	0.00	0.360	−0.107	0.0121	0.00
	(0.00329)	(0.000972)	(0.0000657)	(0.00)	(0.00693)	(0.000221)	(0.00000396)	(0.00)

eTable 6: Summary of the multivariate normal simulation results of the two-stage least squares estimate of the casual exposure effect, $\hat{\beta}_X^{2SLS}$, for true value of 0 and nonlinear $X - Z$ association: Mean (standard deviation) of the partial $R^2_{X|Z}$ of the $X - Z$ association, bias of $\hat{\beta}_X^{2SLS}$, mean of the estimated standard errors, SE, of $\hat{\beta}_X^{2SLS}$, and coverage percentage of the 95% confidence interval for $\hat{\beta}_X^{2SLS}$. Monte Carlo errors in brackets, except for partial $R^2_{X|Z}$.

Full sample/	Moderate Instrument				Strong Instrument			
Selection	partial $R^2_{X\|Z}$	Bias	Mean SE	Coverage	partial $R^2_{X\|Z}$	Bias	Mean SE	Coverage
Full sample	0.0454	0.000196	0.0192	95.3	0.393	0.000110	0.00596	95.3
	(0.00500)	(0.000346)	(0.0000282)	(0.388)	(0.00663)	(0.000107)	(0.00000254)	(0.386)
Completely at random	0.0455	0.000180	0.0249	95.0	0.393	0.000130	0.00766	95.1
	(0.00655)	(0.000448)	(0.0000479)	(0.397)	(0.00866)	(0.000138)	(0.00000426)	(0.394)
on Z	0.0319	−0.000298	0.0312	95.1	0.350	0.0000716	0.00865	95.1
	(0.00555)	(0.000563)	(0.0000724)	(0.395)	(0.00829)	(0.000157)	(0.00000506)	(0.394)
on Z + C	0.0272	−0.174	0.0384	0.00	0.352	−0.0485	0.00877	0.00
	(0.00523)	(0.000785)	(0.000115)	(0.00)	(0.00840)	(0.000162)	(0.00000529)	(0.00)
on X	0.0335	0.00156	0.0317	95.0	0.339	−0.0528	0.00938	0.00
	(0.00573)	(0.000570)	(0.0000719)	(0.397)	(0.00824)	(0.000174)	(0.00000575)	(0.00)
on X + C	0.0340	−0.00962	0.0312	94.4	0.351	−0.0488	0.00906	0.0333
	(0.00572)	(0.000565)	(0.0000698)	(0.421)	(0.00832)	(0.000168)	(0.00000541)	(0.0333)
on X + Z	0.0180	−0.132	0.0486	17.9	0.323	−0.0583	0.00970	0.00
	(0.00445)	(0.000947)	(0.000184)	(0.699)	(0.00822)	(0.000180)	(0.00000618)	(0.00)
on Y	0.0466	0.000692	0.0228	95.2	0.405	0.000225	0.00703	94.7
	(0.00643)	(0.000419)	(0.0000424)	(0.392)	(0.00855)	(0.000129)	(0.00000381)	(0.409)
on X + Y	0.0347	−0.0200	0.0301	90.4	0.365	−0.0503	0.00862	0.00
	(0.00574)	(0.000550)	(0.0000657)	(0.537)	(0.00840)	(0.000159)	(0.00000499)	(0.00)
on Y + Z	0.0313	−0.234	0.0358	0.00	0.375	−0.0542	0.00796	0.00
	(0.00563)	(0.000790)	(0.000105)	(0.00)	(0.00847)	(0.000146)	(0.00000472)	(0.00)

eTable 7: Summary of the multivariate normal simulation results of the two-stage least squares estimate of the casual exposure effect, $\hat{\beta}_X^{2SLS}$, for true value of 1 and linear $X-Z$ association: Mean (standard deviation) of the partial $R^2_{X|Z}$ of the $X-Z$ association, bias of $\hat{\beta}_X^{2SLS}$, mean of the estimated standard errors, SE, of $\hat{\beta}_X^{2SLS}$, and coverage percentage of the 95% confidence interval for $\hat{\beta}_X^{2SLS}$. Monte Carlo errors in brackets, except for partial $R^2_{X|Z}$.

Full sample/	partial $R^2_{X\|Z} = 0.15$				Strong Instrument			
Selection	partial $R^2_{X\|Z}$	Bias	Mean SE	Coverage	partial $R^2_{X\|Z}$	Bias	Mean SE	Coverage
Full sample	0.151<br>(0.00463)	0.000313<br>(0.000304)	0.0168<br>(0.00000603)	95.4<br>(0.382)	0.390<br>(0.00535)	0.000181<br>(0.000160)	0.00884<br>(0.00000201)	95.3<br>(0.385)
Completely at random	0.151<br>(0.00602)	0.000519<br>(0.000390)	0.0216<br>(0.0000101)	95.4<br>(0.382)	0.390<br>(0.00695)	0.000286<br>(0.000205)	0.0114<br>(0.00000341)	95.3<br>(0.385)
on Z	0.130<br>(0.00564)	0.000175<br>(0.000428)	0.0237<br>(0.0000118)	94.9<br>(0.400)	0.350<br>(0.00693)	0.000121<br>(0.000226)	0.0125<br>(0.00000393)	94.8<br>(0.404)
on Z + C	0.125<br>(0.00564)	−0.0782<br>(0.000447)	0.0247<br>(0.0000134)	10.9<br>(0.568)	0.353<br>(0.00698)	−0.0470<br>(0.000227)	0.0125<br>(0.00000406)	3.33<br>(0.328)
on X	0.130<br>(0.00558)	−0.0421<br>(0.000464)	0.0256<br>(0.0000130)	61.3<br>(0.889)	0.348<br>(0.00691)	−0.0429<br>(0.000243)	0.0133<br>(0.00000426)	9.67<br>(0.540)
on X + C	0.142<br>(0.00591)	−0.0382<br>(0.000436)	0.0237<br>(0.0000117)	63.5<br>(0.879)	0.370<br>(0.00690)	−0.0432<br>(0.000229)	0.0126<br>(0.00000391)	6.90<br>(0.463)
on X + Z	0.0989<br>(0.00515)	−0.0651<br>(0.000529)	0.0289<br>(0.0000174)	38.3<br>(0.888)	0.325<br>(0.00696)	−0.0404<br>(0.000249)	0.0136<br>(0.00000458)	15.9<br>(0.668)
on Y	0.142<br>(0.00593)	−0.0811<br>(0.000423)	0.0228<br>(0.0000112)	5.20<br>(0.405)	0.373<br>(0.00693)	−0.0704<br>(0.000223)	0.0121<br>(0.00000374)	0.00<br>(0.00)
on X + Y	0.137<br>(0.00580)	−0.0808<br>(0.000450)	0.0242<br>(0.0000121)	8.90<br>(0.520)	0.362<br>(0.00692)	−0.0688<br>(0.000231)	0.0127<br>(0.00000398)	0.00<br>(0.00)
on Y + Z	0.113<br>(0.00538)	−0.184<br>(0.000496)	0.0270<br>(0.0000157)	0.00<br>(0.00)	0.342<br>(0.00699)	−0.0980<br>(0.000240)	0.0131<br>(0.00000429)	0.00<br>(0.00)

eTable 8: Summary of the multivariate normal simulation results of the two-stage least squares estimator of the casual exposure effect conditional on C, $\hat{\beta}^{2SLS}_{X|C}$, for a linear $X - Z$ association and a causal exposure effect of 1: Mean (standard deviation) of the partial $R^2_{X|Z}$ of the $X - Z$ association, bias of $\hat{\beta}^{2SLS}_{X|C}$, mean of the estimated standard errors, SE, of $\hat{\beta}^{2SLS}_{X|C}$, and coverage percentage of the 95% confidence interval for $\hat{\beta}^{2SLS}_{X|C}$. Monte Carlo errors in brackets, except for partial $R^2_{X|Z}$.

Full sample/	Moderate Instrument				Strong Instrument			
Selection	partial $R^2_{X\|Z}$	Bias	Mean SE	Coverage	partial $R^2_{X\|Z}$	Bias	Mean SE	Coverage
Full sample	0.0508<br>(0.00301)	0.000406<br>(0.000557)	0.0306<br>(0.0000181)	95.3<br>(0.386)	0.438<br>(0.00521)	0.000123<br>(0.000145)	0.00801<br>(0.00000162)	95.3<br>(0.385)
Completely at random	0.0508<br>(0.00390)	0.000937<br>(0.000714)	0.0394<br>(0.0000304)	95.1<br>(0.393)	0.438<br>(0.00678)	0.000274<br>(0.000186)	0.0103<br>(0.00000274)	94.8<br>(0.405)
on Z	0.0431<br>(0.00356)	0.0000655<br>(0.000791)	0.0432<br>(0.0000357)	95.2<br>(0.392)	0.396<br>(0.00679)	0.0000484<br>(0.000207)	0.0113<br>(0.00000315)	95.2<br>(0.390)
on Z + C	0.0468<br>(0.00376)	0.000468<br>(0.000745)	0.0413<br>(0.0000333)	95.5<br>(0.378)	0.417<br>(0.00678)	0.000156<br>(0.000195)	0.0108<br>(0.00000296)	95.4<br>(0.382)
on X	0.0437<br>(0.00368)	−0.0208<br>(0.000846)	0.0462<br>(0.0000395)	93.3<br>(0.456)	0.399<br>(0.00681)	−0.0215<br>(0.000213)	0.0117<br>(0.00000326)	54.6<br>(0.909)
on X + C	0.0474<br>(0.00382)	−0.00859<br>(0.000778)	0.0425<br>(0.0000347)	94.8<br>(0.407)	0.416<br>(0.00677)	−0.0118<br>(0.000202)	0.0111<br>(0.00000307)	82.7<br>(0.691)
on X + Z	0.0209<br>(0.00259)	−0.0743<br>(0.00120)	0.0662<br>(0.0000829)	80.3<br>(0.727)	0.377<br>(0.00693)	−0.0200<br>(0.000221)	0.0120<br>(0.00000347)	60.7<br>(0.892)
on Y	0.0479<br>(0.00384)	−0.0687<br>(0.000760)	0.0411<br>(0.0000332)	60.9<br>(0.891)	0.421<br>(0.00677)	−0.0527<br>(0.000199)	0.0108<br>(0.00000297)	0.333<br>(0.105)
on X + Y	0.0460<br>(0.00374)	−0.0659<br>(0.000801)	0.0436<br>(0.0000358)	67.8<br>(0.853)	0.411<br>(0.00680)	−0.0477<br>(0.000205)	0.0113<br>(0.00000311)	1.20<br>(0.199)
on Y + Z	0.0303<br>(0.00306)	−0.310<br>(0.00102)	0.0556<br>(0.0000640)	0.0333<br>(0.0333)	0.394<br>(0.00686)	−0.0720<br>(0.000210)	0.0115<br>(0.00000324)	0.00<br>(0.00)

eTable 9: Summary of the multivariate normal simulation results of the two-stage least squares estimator of the casual exposure effect conditional on C, $\hat{\beta}^{2SLS}_{X|C}$, for a nonlinear $X - Z$ association and a causal exposure effect of 1: Mean (standard deviation) of the partial $R^2_{X|Z}$ of the $X - Z$ association, bias of $\hat{\beta}^{2SLS}_{X|C}$, mean of the estimated standard errors, SE, of $\hat{\beta}^{2SLS}_{X|C}$, and coverage percentage of the 95% confidence interval for $\hat{\beta}^{2SLS}_{X|C}$. Monte Carlo errors in brackets, except for partial $R^2_{X|Z}$.

Full sample/	Moderate Instrument				Strong Instrument			
Selection	partial $R^2_{X\|Z}$	Bias	Mean SE	Coverage	partial $R^2_{X\|Z}$	Bias	Mean SE	Coverage
Full sample	0.0505	0.000105	0.0157	95.3	0.443	0.0000527	0.00461	95.0
	(0.00528)	(0.000285)	(0.0000224)	(0.386)	(0.00707)	(0.0000834)	(0.00000187)	(0.398)
Completely at random	0.0505	0.000300	0.0203	95.0	0.443	0.000119	0.00593	95.0
	(0.00691)	(0.000367)	(0.0000377)	(0.399)	(0.00914)	(0.000108)	(0.00000313)	(0.397)
on Z	0.0357	−0.000303	0.0255	94.9	0.401	0.00000144	0.00669	94.8
	(0.00591)	(0.000468)	(0.0000572)	(0.402)	(0.00873)	(0.000123)	(0.00000370)	(0.405)
on Z + C	0.0407	0.0000338	0.0234	95.1	0.420	0.0000624	0.00634	95.0
	(0.00615)	(0.000424)	(0.0000478)	(0.394)	(0.00887)	(0.000116)	(0.00000338)	(0.397)
on X	0.0365	0.000362	0.0263	95.5	0.394	−0.0263	0.00704	3.23
	(0.00605)	(0.000476)	(0.0000590)	(0.377)	(0.00882)	(0.000130)	(0.00000395)	(0.323)
on X + C	0.0368	−0.00329	0.0260	95.2	0.403	−0.0195	0.00685	18.9
	(0.00603)	(0.000467)	(0.0000574)	(0.390)	(0.00888)	(0.000127)	(0.00000379)	(0.715)
on X + Z	0.0218	−0.0651	0.0358	55.2	0.379	−0.0300	0.00723	1.23
	(0.00493)	(0.000680)	(0.000116)	(0.908)	(0.00869)	(0.000133)	(0.00000418)	(0.202)
on Y	0.0381	−0.0122	0.0248	92.2	0.416	−0.0289	0.00659	0.667
	(0.00608)	(0.000458)	(0.0000534)	(0.491)	(0.00899)	(0.000121)	(0.00000355)	(0.149)
on X + Y	0.0371	−0.00649	0.0256	94.3	0.407	−0.0291	0.00678	1.10
	(0.00602)	(0.000471)	(0.0000561)	(0.424)	(0.00891)	(0.000126)	(0.00000372)	(0.190)
on Y + Z	0.0281	−0.0844	0.0305	18.7	0.402	−0.0365	0.00680	0.00
	(0.00541)	(0.000586)	(0.0000829)	(0.712)	(0.00888)	(0.000127)	(0.00000377)	(0.00)

eTable 10: Summary of the multivariate normal simulation results of the two-stage least squares estimator of the casual exposure effect conditional on C, $\hat{\beta}^{2SLS}_{X|C}$, for a linear $X-Z$ association and a null causal exposure effect: Mean (standard deviation) of the partial $R^2_{X|Z}$ of the $X-Z$ association, bias of $\hat{\beta}^{2SLS}_{X|C}$, mean of the estimated standard errors, SE, of $\hat{\beta}^{2SLS}_{X|C}$, and coverage percentage of the 95% confidence interval for $\hat{\beta}^{2SLS}_{X|C}$. Monte Carlo errors in brackets, except for partial $R^2_{X|Z}$.

Full sample/	Moderate Instrument				Strong Instrument			
Selection	partial $R^2_{X\|Z}$	Bias	Mean SE	Coverage	partial $R^2_{X\|Z}$	Bias	Mean SE	Coverage
Full sample	0.0508 (0.00301)	0.000406 (0.000557)	0.0306 (0.0000181)	95.3 (0.386)	0.438 (0.00521)	0.000123 (0.000145)	0.00801 (0.00000162)	95.3 (0.385)
Completely at random	0.0508 (0.00390)	0.000937 (0.000714)	0.0394 (0.0000304)	95.1 (0.393)	0.438 (0.00678)	0.000274 (0.000186)	0.0103 (0.00000274)	94.8 (0.405)
on Z	0.0431 (0.00356)	0.0000655 (0.000791)	0.0432 (0.0000357)	95.2 (0.392)	0.396 (0.00679)	0.0000484 (0.000207)	0.0113 (0.00000315)	95.2 (0.390)
on Z + C	0.0468 (0.00376)	0.000468 (0.000745)	0.0413 (0.0000333)	95.5 (0.378)	0.417 (0.00678)	0.000156 (0.000195)	0.0108 (0.00000296)	95.4 (0.382)
on X	0.0437 (0.00368)	−0.0208 (0.000846)	0.0462 (0.0000395)	93.3 (0.456)	0.399 (0.00681)	−0.0215 (0.000213)	0.0117 (0.00000326)	54.6 (0.909)
on X + C	0.0474 (0.00382)	−0.00859 (0.000778)	0.0425 (0.0000347)	94.8 (0.407)	0.416 (0.00677)	−0.0118 (0.000202)	0.0111 (0.00000307)	82.7 (0.691)
on X + Z	0.0209 (0.00259)	−0.0743 (0.00120)	0.0662 (0.0000829)	80.3 (0.727)	0.377 (0.00693)	−0.0200 (0.000221)	0.0120 (0.00000347)	60.7 (0.892)
on Y	0.0510 (0.00388)	0.00101 (0.000659)	0.0366 (0.0000279)	95.6 (0.376)	0.440 (0.00675)	0.000235 (0.000174)	0.00961 (0.00000256)	95.0 (0.397)
on X + Y	0.0479 (0.00384)	−0.0687 (0.000760)	0.0411 (0.0000332)	60.9 (0.891)	0.420 (0.00677)	−0.0537 (0.000199)	0.0108 (0.00000297)	0.267 (0.0942)
on Y + Z	0.0437 (0.00357)	−0.356 (0.000836)	0.0455 (0.0000458)	0.00 (0.00)	0.414 (0.00670)	−0.0879 (0.000196)	0.0106 (0.00000301)	0.00 (0.00)

eTable 11: Summary of the multivariate normal simulation results of the two-stage least squares estimator of the casual exposure effect conditional on C, $\hat{\beta}^{2SLS}_{X|C}$, for a nonlinear $X - Z$ association and a null causal exposure effect: Mean (standard deviation) of the partial $R^2_{X|Z}$ of the $X - Z$ association, bias of $\hat{\beta}^{2SLS}_{X|C}$, mean of the estimated standard errors, SE, of $\hat{\beta}^{2SLS}_{X|C}$, and coverage percentage of the 95% confidence interval for $\hat{\beta}^{2SLS}_{X|C}$. Monte Carlo errors in brackets, except for partial $R^2_{X|Z}$.

Full sample/	Moderate Instrument				Strong Instrument			
Selection	partial $R^2_{X\|Z}$	Bias	Mean SE	Coverage	partial $R^2_{X\|Z}$	Bias	Mean SE	Coverage
Full sample	0.0512	0.000105	0.0156	95.3	0.443	0.0000527	0.00461	95.0
	(0.00530)	(0.000283)	(0.0000221)	(0.386)	(0.00707)	(0.0000834)	(0.00000187)	(0.398)
Completely at random	0.0512	0.000299	0.0202	95.0	0.443	0.000119	0.00593	95.0
	(0.00694)	(0.000365)	(0.0000372)	(0.399)	(0.00914)	(0.000108)	(0.00000313)	(0.397)
on Z	0.0363	−0.000298	0.0253	94.9	0.401	0.00000144	0.00669	94.8
	(0.00594)	(0.000464)	(0.0000562)	(0.403)	(0.00873)	(0.000123)	(0.00000370)	(0.405)
on Z + C	0.0413	0.0000353	0.0232	95.1	0.420	0.0000624	0.00634	95.0
	(0.00618)	(0.000421)	(0.0000470)	(0.394)	(0.00887)	(0.000116)	(0.00000338)	(0.397)
on X	0.0370	0.000546	0.0262	95.5	0.393	−0.0268	0.00705	3.03
	(0.00607)	(0.000473)	(0.0000581)	(0.378)	(0.00881)	(0.000130)	(0.00000396)	(0.313)
on X + C	0.0373	−0.00344	0.0258	95.0	0.404	−0.0189	0.00684	20.5
	(0.00606)	(0.000463)	(0.0000565)	(0.397)	(0.00887)	(0.000127)	(0.00000377)	(0.737)
on X + Z	0.0223	−0.0649	0.0355	54.7	0.380	−0.0292	0.00720	1.60
	(0.00496)	(0.000672)	(0.000113)	(0.909)	(0.00871)	(0.000133)	(0.00000415)	(0.229)
on Y	0.0515	0.000532	0.0191	95.6	0.447	0.000113	0.00563	94.7
	(0.00674)	(0.000348)	(0.0000341)	(0.374)	(0.00901)	(0.000103)	(0.00000291)	(0.409)
on X + Y	0.0386	−0.0125	0.0247	92.0	0.416	−0.0289	0.00659	0.667
	(0.00611)	(0.000454)	(0.0000526)	(0.494)	(0.00899)	(0.000121)	(0.00000355)	(0.149)
on Y + Z	0.0401	−0.149	0.0251	0.00	0.428	−0.0336	0.00608	0.0333
	(0.00626)	(0.000533)	(0.0000607)	(0.00)	(0.00897)	(0.000111)	(0.00000335)	(0.0333)

eTable 12: Summary of the multivariate normal simulation results of the inverse probability weighted two-stage least squares estimate of the casual exposure effect, $\hat{\beta}_X^{IPW,2SLS}$, for a true value of 1 and linear $X - Z$ association: Mean (standard deviation) of the partial $R^2_{X|Z}$ of the $X - Z$ association, bias of $\hat{\beta}_X^{IPW,2SLS}$, mean of the estimated standard errors, SE, of $\hat{\beta}_X^{IPW,2SLS}$, and coverage percentage of the 95% confidence interval for $\hat{\beta}_X^{IPW,2SLS}$. Monte Carlo errors in brackets, except for partial $R^2_{X|Z}$.

	Moderate Instrument				Strong Instrument			
Selection	partial $R^2_{X\|Z}$	Bias	Mean SE	Coverage	partial $R^2_{X\|Z}$	Bias	Mean SE	Coverage
Z	0.0384	−0.000238	0.0569	96.1	0.350	0.00000311	0.0154	95.6
	(0.00338)	(0.00102)	(0.000257)	(0.355)	(0.00693)	(0.000276)	(0.0000513)	(0.373)
Z + C	0.0322	0.000201	0.0521	95.6	0.353	0.000122	0.0142	95.8
	(0.00317)	(0.000938)	(0.0000924)	(0.374)	(0.00698)	(0.000257)	(0.0000260)	(0.366)
X	0.0382	0.000426	0.0474	95.1	0.348	−0.0000183	0.0140	95.1
	(0.00345)	(0.000864)	(0.0000608)	(0.394)	(0.00691)	(0.000254)	(0.0000212)	(0.394)
X + C	0.0425	0.000245	0.0480	94.9	0.370	0.0000462	0.0138	95.4
	(0.00364)	(0.000883)	(0.0000650)	(0.402)	(0.00690)	(0.000249)	(0.0000199)	(0.384)
X + Z	0.0163	−0.000217	0.0527	95.3	0.325	0.0000371	0.0147	95.3
	(0.00229)	(0.000960)	(0.0000786)	(0.386)	(0.00696)	(0.000269)	(0.0000227)	(0.388)
Y	0.0425	0.0000387	0.0528	94.9	0.373	0.0000751	0.0148	95.0
	(0.00366)	(0.000966)	(0.0000921)	(0.402)	(0.00693)	(0.000269)	(0.0000312)	(0.398)
X + Y	0.0406	0.000115	0.0505	94.6	0.362	−0.0000451	0.0144	95.5
	(0.00355)	(0.000928)	(0.0000796)	(0.411)	(0.00692)	(0.000257)	(0.0000266)	(0.378)
Y + Z	0.0241	0.0000617	0.0552	94.8	0.343	−0.000502	0.0153	94.9
	(0.00278)	(0.00132)	(0.000162)	(0.532)	(0.00742)	(0.00122)	(0.000113)	(1.74)

eTable 13: Summary of the multivariate normal simulation results of the inverse probability weighted two-stage least squares estimate of the casual exposure effect, $\hat{\beta}_X^{IPW,2SLS}$, for true value of 1 and nonlinear $X - Z$ association: Mean (standard deviation) of the partial $R^2_{X|Z}$ of the $X - Z$ association, bias of $\hat{\beta}_X^{IPW,2SLS}$, mean of the estimated standard errors, SE, of $\hat{\beta}_X^{IPW,2SLS}$, and coverage percentage of the 95% confidence interval for $\hat{\beta}_X^{IPW,2SLS}$. Monte Carlo errors in brackets, except for partial $R^2_{X|Z}$.

	Moderate Instrument				Strong Instrument			
Selection	partial $R^2_{X\|Z}$	Bias	Mean SE	Coverage	partial $R^2_{X\|Z}$	Bias	Mean SE	Coverage
Z	0.0314	−0.000852	0.0352	95.5	0.350	−0.000101	0.0103	94.9
	(0.00552)	(0.000638)	(0.000126)	(0.377)	(0.00829)	(0.000188)	(0.0000138)	(0.400)
Z + C	0.0267	−0.00101	0.0326	95.8	0.352	−0.0000797	0.00984	95.4
	(0.00520)	(0.000583)	(0.0000965)	(0.366)	(0.00840)	(0.000177)	(0.0000177)	(0.382)
X	0.0330	−0.0110	0.0589	90.5	0.339	−0.00187	0.0171	91.1
	(0.00571)	(0.00116)	(0.000353)	(0.535)	(0.00826)	(0.000373)	(0.000200)	(0.521)
X + C	0.0335	−0.00805	0.0509	90.6	0.350	−0.00152	0.0151	91.6
	(0.00569)	(0.00100)	(0.000254)	(0.532)	(0.00833)	(0.000327)	(0.000138)	(0.506)
X + Z	0.0177	−0.0134	0.0752	89.2	0.322	−0.00167	0.0173	91.4
	(0.00442)	(0.00154)	(0.000523)	(0.566)	(0.00822)	(0.000378)	(0.000187)	(0.511)
Y	0.0343	−0.00692	0.0470	91.7	0.365	−0.000946	0.0137	92.8
	(0.00571)	(0.000927)	(0.000222)	(0.504)	(0.00840)	(0.000283)	(0.000122)	(0.471)
X + Y	0.0334	−0.00894	0.0521	90.7	0.354	−0.00138	0.0147	92.2
	(0.00568)	(0.00102)	(0.000265)	(0.530)	(0.00836)	(0.000319)	(0.000139)	(0.490)
Y + Z	0.0233	−0.00876	0.0526	91.3	0.348	−0.00112	0.0141	92.7
	(0.00492)	(0.00103)	(0.000273)	(0.515)	(0.00838)	(0.000292)	(0.000121)	(0.475)

eTable 14: Distribution of the instrument, exposure and two outcomes in the applied example dataset from the UK Biobank study.

No. participants	Turned 15 years after policy introduced	Left school aged 16 years or older	Percentage of ever smokers	Percentage of current smokers
1967	No	No	57.4%	23.5%
9273	No	Yes	37.1%	9.14%
328	Yes	No	65.3%	29.4%
10570	Yes	Yes	39.2%	11.4%

eTable 15: Values of the selection model's parameters for 9 selection mechanisms, including selection completely at random (SCAR), used in the applied example simulation study.

Selection is/ depends on	η_0	η_Z	η_U	η_X	η_Y
SCAR	0.425	0	0	0	0
Z	−0.390	1.81	0	0	0
$Z + U$	0.0500	0.900	0.900	0	0
X	−1.20	0	0	2.10	0
$X + U$	−0.270	0	1.00	1.00	0
$X + Z$	−1.12	1.27	0	1.27	0
Y	−0.170	0	0	0	2.16
$X + Y$	−1.440	0	0	1.75	1.75
$Y + Z$	−0.680	1.42	0	0	1.42

eTable 16: Summary of the applied example simulation results of the two-stage least squares estimate of the casual exposure effect, $\hat{\beta}_X^{2SLS}$: Mean (standard deviation) of the partial $R^2_{X|Z}$ of the $X - Z$ association, bias of $\hat{\beta}_X^{2SLS}$, mean of the estimated standard errors, SE, of $\hat{\beta}_X^{2SLS}$, coverage percentage of the 95% confidence interval for $\hat{\beta}_X^{2SLS}$, mean (standard deviation) of the coefficients for the linear regressions of Z given U Monte Carlo errors in brackets, except for partial $R^2_{X|Z}$.

Full sample/	partial $R^2_{X\|Z} = 0.056$			
Selection	partial $R^2_{X\|Z}$	Bias	Mean SE	Coverage
Full sample	0.0560<br>(0.00309)	0.000763<br>(0.000610)	0.0336<br>(0.0000180)	95.5<br>(0.378)
Completely at random	0.0560<br>(0.00397)	0.00127<br>(0.000791)	0.0432<br>(0.0000299)	95.4<br>(0.384)
on Z	0.0565<br>(0.00458)	0.000244<br>(0.000834)	0.0457<br>(0.0000364)	95.3<br>(0.386)
on Z + U	0.0739<br>(0.00482)	−0.0905<br>(0.000688)	0.0376<br>(0.0000235)	32.5<br>(0.855)
on X	0.0258<br>(0.00276)	0.113<br>(0.00172)	0.0946<br>(0.000106)	78.1<br>(0.755)
on X + U	0.0515<br>(0.00384)	−0.00681<br>(0.000871)	0.0480<br>(0.0000353)	95.1<br>(0.393)
on X + Z	0.0185<br>(0.00259)	0.115<br>(0.00177)	0.0969<br>(0.000138)	78.4<br>(0.751)
on Y	0.0590<br>(0.00412)	−0.00171<br>(0.000782)	0.0434<br>(0.0000295)	95.1<br>(0.395)
on X + Y	0.0371<br>(0.00330)	−0.103<br>(0.00124)	0.0671<br>(0.0000603)	64.8<br>(0.872)
on Y + Z	0.0662<br>(0.00468)	−0.475<br>(0.000810)	0.0441<br>(0.0000336)	0.00<br>(0.00)

eTable 17: Summary of the applied example simulation results of the of the inverse probability weighted two-stage least squares estimate of the casual exposure effect, $\hat{\beta}_X^{IPW,2SLS}$: Mean (standard deviation) of the partial $R^2_{X|Z}$ of the $X - Z$ association, bias of $\hat{\beta}_X^{IPW,2SLS}$, mean of the estimated standard errors, SE, of $\hat{\beta}_X^{IPW,2SLS}$, coverage percentage of the 95% confidence interval for $\hat{\beta}_X^{IPW,2SLS}$, mean (standard deviation) of the coefficients for the linear regressions of Z given U Monte Carlo errors in brackets, except for partial $R^2_{X|Z}$.

	partial $R^2_{X\|Z} = 0.056$			
Selection	partial $R^2_{X\|Z}$	Bias	Mean SE	Coverage
on Z	0.0561<br>(0.00434)	0.000244<br>(0.000834)	0.0458<br>(0.0000363)	95.4<br>(0.381)
on Z + U	0.0560<br>(0.00416)	0.000427<br>(0.000818)	0.0454<br>(0.0000344)	95.5<br>(0.378)
on X	0.0562<br>(0.00581)	0.000314<br>(0.000913)	0.0495<br>(0.0000505)	94.6<br>(0.413)
on X + U	0.0561<br>(0.00448)	0.00105<br>(0.000821)	0.0453<br>(0.0000355)	95.5<br>(0.378)
on X + Z	0.0560<br>(0.00465)	0.00101<br>(0.000879)	0.0476<br>(0.0000389)	94.9<br>(0.400)
on Y	0.0561<br>(0.00410)	0.000786<br>(0.000740)	0.0411<br>(0.0000296)	95.4<br>(0.382)
on X + Y	0.0561<br>(0.00543)	0.000921<br>(0.000819)	0.0442<br>(0.0000429)	95.0<br>(0.399)
on Y + Z	0.0561<br>(0.00424)	0.00106<br>(0.000725)	0.0427<br>(0.0000328)	96.9<br>(0.316)
